# Evaluating a potential model to analyze the function of the gut microbiota of the giant panda

**DOI:** 10.3389/fmicb.2022.1086058

**Published:** 2022-12-20

**Authors:** Wenping Zhang, Junjin Xie, Shan Xia, Xueyang Fan, Stephan Schmitz-Esser, Benhua Zeng, Lijun Zheng, He Huang, Hairui Wang, Jincheng Zhong, Zhihe Zhang, Liang Zhang, Mingfeng Jiang, Rong Hou

**Affiliations:** ^1^Chengdu Research Base of Giant Panda Breeding, Chengdu, Sichuan, China; ^2^Sichuan Key Laboratory of Conservation Biology for Endangered Wildlife, Chengdu, Sichuan, China; ^3^Qinghai-Tibet Plateau Research Institute, Southwest Minzu University, Chengdu, Sichuan, China; ^4^College of Chemistry and Life Science, Chengdu Normal University, Chengdu, Sichuan, China; ^5^Department of Animal Science, Iowa State University, Ames, IA, United States; ^6^Department of Infectious Diseases, Southwest Hospital, Army Medical University (Third Military Medical University), Chongqing, China; ^7^Sichuan Academy of Giant Panda, Chengdu, Sichuan, China

**Keywords:** giant panda, horse, germ-free mice, 16S rRNA gene sequencing, blood metabolites

## Abstract

To contribute to the conservation of endangered animals, the utilization of model systems is critical to analyze the function of their gut microbiota. In this study, the results of a fecal microbial transplantation (FMT) experiment with germ-free (GF) mice receiving giant panda or horse fecal microbiota showed a clear clustering by donor microbial communities in GF mice, which was consistent with the results of blood metabolites from these mice. At the genus level, FMT re-established approximately 9% of the giant panda donor microbiota in GF mice compared to about 32% for the horse donor microbiota. In line with this, the difference between the panda donor microbiota and panda-mice microbiota on whole-community level was significantly larger than that between the horse donor microbiota and the horse-mice microbiota. These results were consistent with source tracking analysis that found a significantly higher retention rate of the horse donor microbiota (30.9%) than the giant panda donor microbiota (4.0%) in GF mice where the microbiota remained stable after FMT. Further analyzes indicated that the possible reason for the low retention rate of the panda donor microbiota in GF mice was a low relative abundance of *Clostridiaceae* in the panda donor microbiota. Our results indicate that the donor microbiota has a large effect on GF mice microbiota after FMT.

## Introduction

Gut microbiomes, the consortia of microorganisms that inhabit the animal gut, are highly specialized microbial communities (Derrien and van Hylckama Vlieg, 2015) and play a key role in animal health, physiology, and nutrition (Wei et al., 2018). Thus, it is crucial to analyze the gut microbiota of giant pandas (*Ailuropoda melanoleuca*) to find novel ways to conserve this endangered flagship species. Working with endangered species can often be challenging because of the limitations of doing invasive work and sampling techniques. Therefore, animal model systems may be essential to study the microbiota of endangered species, such as the giant panda.

Many strains of murine models are inbred and the availability of genetically modified lines facilitates research aiming at elucidating the interaction between the gut microbiome, host genetic background and disease (Carvalho et al., 2012). Moreover, germ-free (GF) mice have no microbiota and can serve as a good model to analyze the relationship between gut microbiota and phenotype with no influence of the mice microbiota (Goodman et al., 2011). Thus, GF mice models have been extensively employed for exploring evidence from human studies and animal models that links intestinal microbiota dysbiosis with a broad-range of immune, metabolic, and neurodevelopmental disorders (Li et al., 2014). This includes irritable bowel syndrome (IBS) (Weingarden and Vaughn, 2017), obesity (Ley et al., 2006; Turnbaugh et al., 2009), schizophrenia (Zhu et al., 2019), and others.

The success of GF mice in human microbiome research has attracted attention to determine whether GF mice could also be used as a model to analyze the function of giant panda fecal bacteria. The giant panda specializes in bamboo eating but harbors a typical carnivorous digestive system and a gut microbial community that is more similar to their carnivorous relatives (i.e., other bears) (Xue et al., 2015). A number of previous studies showed that *Firmicutes* and *Proteobacteria* were the main bacterial phyla of the gut microbiota of giant pandas (Zhu et al., 2011; Xue et al., 2015; Zhang et al., 2018; Guo et al., 2019). In contrast, the main gut bacteria of humans and mice belong to *Firmicutes* and *Bacteroidetes* (Turnbaugh et al., 2009; Zhou et al., 2019). Zhou et al. (2019) found that many species of the *Bacteroidetes* phylum from human fecal samples were successfully colonizing mice, while multiple donor genera from the *Firmicutes* phylum did not colonize the mouse gut. Similar to the human and mouse microbiota, the main bacteria of the herbivorous horse were also *Firmicutes* and *Bacteroidetes* (Mienaltowski et al., 2020). However, no publications were reported to analyze whether the gut bacteria of horses could successfully colonize mice like members of the human gut microbiota. So, we used the horse as a control to compare the ratio of re-establishing of gut bacteria between giant panda and horse donors into mice.

Moreover, the highly complex and diverse microbial consortium in the mammalian gastrointestinal tract maintains a mutualistic relationship with the host and is influenced in many ways, such as diet, genetics, environment, mode of birth, infant feeding, lifestyle, medication, and others (De Filippo et al., 2010; David et al., 2014; Rooks and Garrett, 2016). In general, more closely phylogenetic related host species have more similar microbiomes (Brucker and Bordenstein, 2012; Brooks et al., 2016; Zhang et al., 2016), and microbiome traits have some level of statistically significant heritability (Grieneisen et al., 2021). Extreme environment can surpass phylogenetic to drive the convergent evolution of gut microbiome. For example, high-altitude drives the convergent evolution of alpha diversity and indicator microbiota in the gut microbiomes of ungulates (Zhang et al., 2016). Furthermore, most mammalian microbiomes are strongly correlated with gut physiology and diet, such as in the convergent evolution of myrmecophagy in mammals (Delsuc et al., 2014), and the convergent evolution of gut microbiomes in bamboo-eating species (Xue et al., 2015), as well as blood feeding in birds and mammals (Song et al., 2019). Some studies have shown that the influence of host species on the structure and function of the gut microbiota is much stronger than that of the dietary niche such as in primates (Perofsky et al., 2018; Amato et al., 2019) and the American pika (Galbreath et al., 2009). In mammals, Song et al. (2020) identified a strong correlation of gut microbiota with both diet and phylogeny. However, in birds, Song et al. (2020) found the gut microbiota in general to be only weakly correlated with host phylogeny and not associated with host diet despite diet varying widely among bird lineages. These studies have often focused on multiple species within a season (Muegge et al., 2011), or one species in different environments (Rothschild et al., 2018), and GF mice can be a good model to analyze the factors under the same environmental conditions. Thus, we also aimed to compare the effect on diet and microbial donor species following the transplantation of the different fecal microbiota of giant pandas and horses into GF mice.

In addition, changes in the gut microbiota will also affect the metabolism of the host, which will also affect the gut microbiota (Wei et al., 2018). Many studies indicate that the gut microbiota influences the development of metabolic syndromes (Turnbaugh et al., 2006; Cho et al., 2012). Indeed, the gut microbiota promotes intestinal epithelial barrier integrity, the development of the immune system, and confers protection against pathogen colonization (Round and Mazmanian, 2009; Pickard et al., 2017). Furthermore, the intestinal microbiota can influence cellular processes at sites distant from the intestine (Schroeder and Bäckhed, 2016). Thus, combined analysis of the microbiome and the metabolome has been suggested as a highly promising approach to evaluate host-microbiome interactions (Turnbaugh and Gordon, 2008).

In this study, we aimed to determine how the giant panda’s microbiota survives in and colonizes GF mice. We used horse microbiota as a control to investigate whether the horse microbiota shows a similar establishment in GF mice as the giant panda microbiota, and to characterize the diversity and composition of the gut microbiota of GF mice colonized with two distinct fecal communities from horse and giant panda as well as blood metabolic changes of these GF mice after fecal microbial transplantation (FMT).

## Materials and methods

### Fecal microbial transplantation analysis and samples

Fecal samples from two species (panda: 5–8 year-old captive giant pandas housed in the Chengdu research base of giant panda breeding; horse: 5–8 year-old captive Chinese miniature ponies kept in the Chengdu Zoo) were used as donors for microbiota transfer experiments. Each species included two individuals (one male and one female) and no antibiotics were fed to all individuals in 2 months prior to sampling.

The fecal samples were freshly collected under anaerobic conditions and kept in an anaerobic chamber at 4°C in a sterile container until processing in the lab within half an hour. Each fecal sample (~100 g) was suspended in sterile phosphate buffer saline (PBS) (3–4 times volume of stool pool of the corresponding group) and vortexed for 5 min followed by sedimentation for 5 min to allow bigger particles to settle to the bottom of the tube. The supernatants of individual fecal samples of each donor species were pooled, and their depositions (cell pellets) were collected by centrifugation at 9,000 g for 5 min. Then, the depositions were washed three times by resuspension and homogenized with 20 ml of sterile PBS and centrifugation at 9,000 g for 5 min. The cell pellets were homogenized and resuspended in sterile 1x PBS solution resulting in a final concentration of 10^9^ bacteria per milliliter determined by hemocytometer counts.

**Figure 1:**
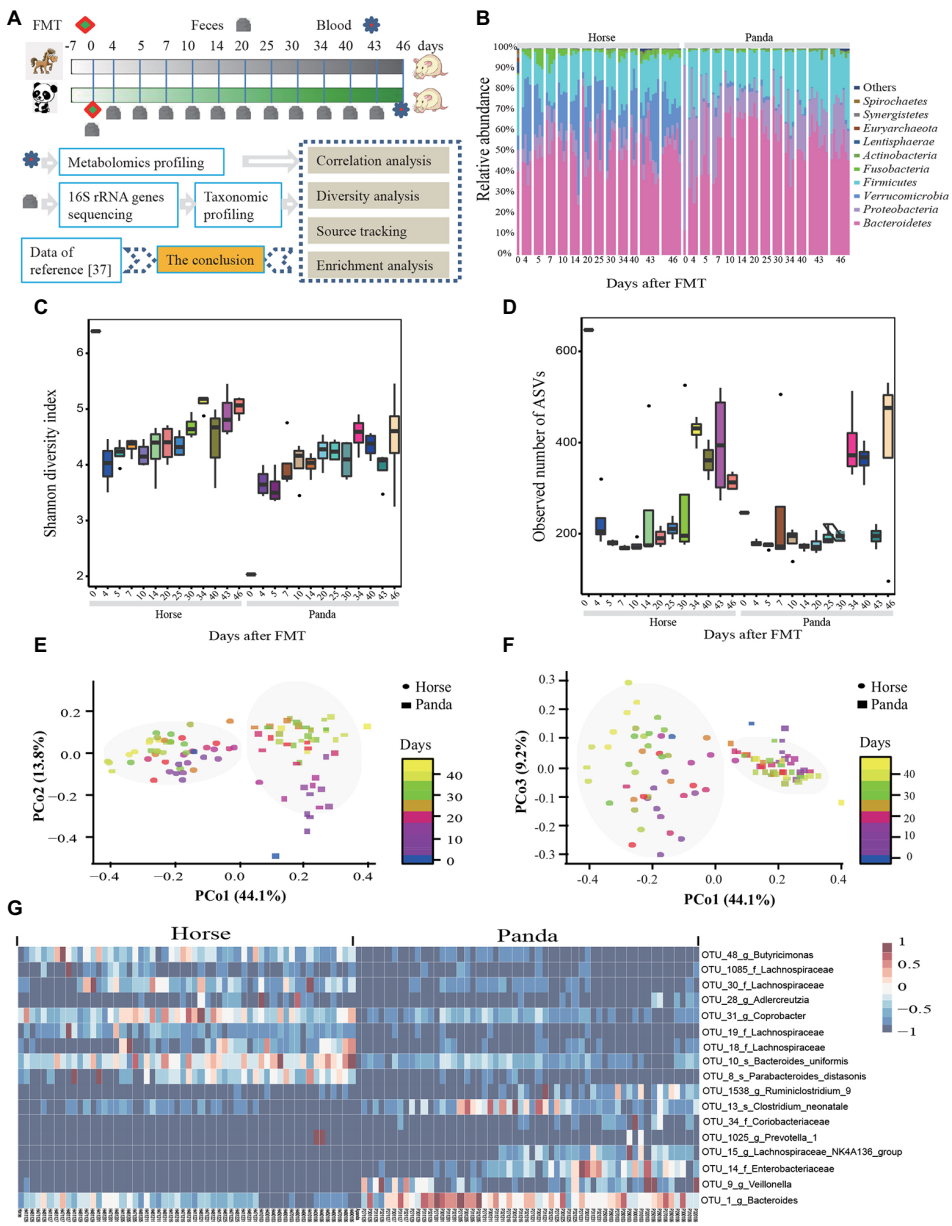
The composition and diversity of gut microbiota in germ-free mice. The experimental design of this study is shown in **(A)**. The conclusion was checked by comparing the data to the recent study by Huang et al. (2022). The composition and diversity included the relative abundance of bacteria of the top ten phyla **(B)**, Shannon diversity index **(C)**, the observed number of ASVs **(D)**, the PCoA plot with PCo1 and PCo2 **(E)**, the PCoA plot with PCo1 and PCo3 **(F)**, and the heat map of the 17 ASV-level phylotypes identified as key variables for differentiation gut microbiota structure between panda-mice and horse-mice **(G)**. Day 0 represents the donor microbiota. Each vertical column denotes one sample. PCoA plots were generated using Bray-Curtis distances of 16S rRNA gene sequences showing the mice microbiota separated by donor in the first axis and shifted with colonized time in the second and third axis and the percentage of variation explained by the plotted principal coordinates is indicated on the axes. Each point corresponds to a community differentiated by shape and collection time by color. An FDR-corrected Wilcoxon rank-sum test was used to determine significance and shown in Supplementary Tables S2–S3 for Shannon diversity **(C)** and the observed number of ASVs **(D)** (*p < 0.05, **p < 0.01, and ***p < 0.001).

GF inbred Balb/c mice (4 weeks old) were housed in 2 gnotobiotic isolators (one for panda-mice and another for horse-mice; Figure 1A) where they were maintained on a strict 12 l: 12D 24 h cycle. A week prior to colonization, mice were fed a standard autoclaved polysaccharide-rich chow diet *ad libitum* (12% cellulose, 12% corn gluten meal, 1.1% soybean oil, and 1.2% CaHPO₄ in basic diet of mice) and divided into two groups with the same sex (female), age and weight. GF status was verified regularly by anaerobic culturing in addition to PCR targeting bacterial 16S rRNA gene. Each mouse was subsequently colonized with a total 1 ml fecal communities by gastric gavage twice (500 μl/time), with an interval of 12 h between procedures. Mice colonized with these two communities were maintained on the same diet (*n* = 8 mice/community, *n* = 16).

Body weight and chow consumption of GF mice were monitored every day. Fresh feces were collected from each mouse at a fixed time every day immediately after the observed excretion of mouse feces and stored at-80°C (Supplementary Table S1). Blood was collected under urethane anesthesia by intraperitoneal injection at the end of the experiment. Serum was separated and stored at-80°C until analysis.

All experimental procedures were implemented according to the guidelines and regulations approved by the Institutional Animal Care and Use Committee (IACUC) at Chengdu Research Base of Giant Panda Breeding (2019015).

### Amplicon sequencing and data processing

Fecal DNA was extracted with the DNA Stool Mini Kit (QIAGEN, Germany) according to the procedure described previously by Zhang et al. (2018) and the DNA concentrations of each sample were adjusted to 50 ng/μL for subsequent 16S rRNA gene sequencing. The V3-V4 region of the bacterial 16S rRNA gene (Kozich et al., 2013) was amplified by a polymerase chain reaction with a 6-bp barcode unique to each sample for the paired primer (forward primer: CTACGGGNGGCWGCAG; reverse primer: GACTACHVGGGTATCTAATCC; Wu et al., 2017; Wang et al., 2022). The PCR products were then pooled and sequenced using the Illumina MiSeq PE-250 platform.

The paired-end fastq files for every individual and the sample information for all individuals following the standard operating protocol and analyzed and assembled using the software package Qiime2 version 2018.11^1^ (Bolyen et al., 2019). The DADA2 plugin in QIIME2 was used for denoise and quality filter reads, removing chimeras, singletons and replicating sequences. To ensure even sequencing depth across samples, 30,000 sequences per sample were randomly subsampled for analysis of bacterial communities and samples with fewer than 30,000 sequences were omitted. After the “Split libraries FASTQ” step, we used a conventional reference-based FEATURE picking strategy against the SILVA reference database v138 (Pruesse et al., 2007) to cluster our 16S rRNA gene sequences into Features with “Pick closed-reference Features” command following the “Defaults” parameter set. Nonbacterial ASVs and sequences identified as chloroplasts and mitochondria were excluded from the data set. After removing low abundance (minimum count = 4 and prevalence in samples = 20%) features, the BIOM-formatted FEATURE table was used to compare species richness and diversity among samples.

GF mice who received the horse donor microbiota were indicated as “horse-mice” and mice who received the panda donor microbiota were indicated as “panda-mice” throughout the manuscript. Bray–Curtis similarity indices and clustering patterns among samples were visualized using principal coordinates analysis (vegan package, R software, version 3.0.2). Permutational multivariate analysis of variance (PERMANOVA) was performed to test whether the gut microbiota structure was significantly different based on 999 permutations in the R “vegan” package. The Mann–Whitney test and paired sample Wilcoxon signed rank test were used for univariate statistical analysis such as α-diversity analysis between horse-mice and panda-mice.

To compare the stability of transplanted microbiota between different honors in the GF mice, we tracked the relative abundance of bacteria at the genus level. All the genus were divided into two groups, the “increased group” and the “decreased group,” by comparing their relative abundance in the original microbiota to mice from the last day of this study despite the donors. Genera with higher abundance in the end of study were considered as the “increased group” and vice versa. Then, we tracked the changes of the total relative abundance of these two groups over this experiment in panda-mice and horse-mice. Average values and standard deviations of relative abundance were calculated at each time point and compared between groups.

To identify the bacterial taxa that can characterize horse and panda groups, we used the random forest model in R (R package ‘Random Forest’, ntree = 1,000) with default parameters (Knights et al., 2011a) and LEfSe using the Huttenhower Galaxy Server.^2^ We formed the dataset and set 0.05 as the value of p threshold in the factorial Kruskal-Wallis test and set 2.0 as the threshold for the LDA score for the LEfSe test. SourceTracker (version 0.9.1) was used to conduct the source tracking analysis for original microbial community in GF mice in R. SourceTracker is a Bayesian method software that estimates the proportion of source microbial composition in a tested sample based on the 16S rRNA gene sequencing data (Knights et al., 2011b). It has been used for tracking the chronological change of the proportion of community of five different sources in the environment (Staley et al., 2018), assessing transfer characteristics (Zhu et al., 2021), and investigating attribute contamination from a variety of fecal source (Sharma et al., 2019). In this study, ASV profile at the genus level was used in this analysis. The depth of rarefication was set at 1000. We set the original microbial community from panda donor or horse donor feces as the “source” and microbial communities in corresponding panda-mice or horse-mice at each time point as the “sink.” Then, we used the Source Tracker to estimate the proportion of the original microbial community remained in the GF mice at each time point to observe the stability of the microbiota after being transplanted. Significance test for the retention proportions of source tracking analysis or Bray-Curtis distances between each time point or groups was performed by applying the One-way ANOVA (ANalysis of VAriance) with post-hoc Tukey HSD (Honestly Significant Difference) by “glht” function in “multicomp” package (Hothorn et al., 2008). Comparison of the retention proportions between panda and horse groups were done with *t*-test. The online tool ImageGP^3^ was used for the data visualization (Tong et al., 2022).

### Metabolomics profiling

Untargeted metabolomic profiles of serum samples of the 16 GF mice were used to measure polar metabolites with LC–MS/MS system on a Waters ACQUITY UPLC HSS T3 column and an Agilent 6,460 triple quadrupole mass spectrometer (Agilent Technologies) by Novogene, Inc., China.

Positive/negative ionization modes were used for the mass spectrometric settings following the methods by Zhang et al., (2017) and the raw data from mass spectrometers were processed using the Progenesis QI software (NonLinear Dynamics). To ensure a high quality of the dataset, control and curation processes were subsequently used to ensure true chemical assignment and remove artifacts and background noise. The bioinformatics program XCMS^4^ (Mahieu et al., 2016) was used for peak finding, filtering, alignment, matching, and identification, then a data matrix consisting of the retention time, m/z value and peak area was obtained and normalized to the total peak area of each chromatogram. Last, the normalized data was imported into the SIMCA-P 14.0 software package (Umetrics AB, Umea, Sweden) for multivariate statistical analysis.

The online tools MetaboAnalyst 3.0^5^ (Montréal, QC, Canada) (Xia et al., 2015), HMDB^6^ and the KEGG database^7^ (Kanehisa and Goto, 2000) were used to investigate the related biochemical pathways and illustrate their connection.

PLS-DA (partial latent structures-discriminant analysis) was applied to explore the metabolite differences between horse-mice and panda-mice following the combination of the VIP > 1 and the *p* < 0.05 from two-tailed *t*-test on the normalized peak intensities. Fold changes (FC) were calculated as a binary logarithm of the average normalized peak area ratio between the two groups and a threshold of FC > 1.2 or < 0.833 was also used to identify the significant different metabolites between panda-mice and horse-mice.

In addition, the correlation between bacterial communities and metabolites was determined by spearman rank correlation coefficient in psych of R.

## Results

### Microbiota composition of panda–mice and horse–mice pairs

Eight GF mice per donor were used for FMT and 113 fecal samples were collected for 16S rRNA gene analysis from 13 time points (Figure 1A). After quality filtering and assembly, 8,916,648 16S rRNA gene sequences were obtained that ranged from 50,362 to 93,902 reads and were grouped into 2,344 Amplicon Sequencing Variants (ASVs) of which 1,354 ASVs belonged to the panda and 1,834 ASVs belonged to the horse (Supplementary Table S1). Across all 16S rRNA gene samples, five phyla were the main bacteria with relative abundances of more than 1% (Figure 1B). Proteobacteria (79.62%), Bacteroidetes (12.07%), and Firmicutes (7.73%) were the main phyla of the giant panda donors, which was consistent with previous research about giant pandas (Zhu et al., 2011; Xue et al., 2015; Zhang et al., 2018; Huang et al., 2022). Similar to giant pandas, the three phyla were also the main phyla of the horse donors but the relative abundance of Bacteroidetes (40.44%) was higher than that of Proteobacteria (10.38%) (Figure 1B), which was in agreement with other studies analyzing the horse microbiota (Mienaltowski et al., 2020).

From gavage to colonization in the intestinal tract of GF mice, *Bacteroidetes* had the highest relative abundance with 53.66%, followed by Firmicutes (20.26%), Proteobacteria (12.90%), Verrucomicrobia (10.72%), and Fusobacteria (1.55%) (Figure 1B), and a big difference of relative abundances at phylum, family and genus level between panda-mice and horse-mice was found (Figure 1B; Supplementary Figure S1). At family level, Bacteroidaceae (40.81%), Enterobacteriaceae (16.78%), Porphyromonadaceae (10.90%), Lachnospiraceae (7.68%), and Alcaligenaceae (6.70%) were the top five families for panda-mice (Supplementary Figure S1). Whereas the top families for horse-mice included Bacteroidaceae (35.33%), Verrucomicrobiaceae (19.31%), Porphyromonadaceae (12.97%), Lachnospiraceae (11.49%), and Enterobacteriaceae (3.40%) (Supplementary Figure S1). At the genus level, 12 genera had more than 1% relative abundance for horse-mice, such as *Bacteroides*, *Akkermansia*, *Parabacteroides*, and so on. Nine genera had more than 1% relative abundance in the panda-mice, which included *Bacteroides*, *Escherichia-Shigella*, *Parabacteroides*, and so on (Supplementary Figure S1).

The observed number of species ranged from 104 to 749 among all samples (Supplementary Table S1). The donor horses’ microbiota had the highest α-diversity and the donor giant pandas’ microbiota had the lowest α-diversity among all samples (Figures 1C,D; Supplementary Tables S1–S3), which is consistent with Xue et al. (2015) who also showed that giant pandas had low gut microbiota diversity compared to published microbiota datasets from 54 mammalian species. Panda-mice displayed significantly lower α-diversity, such as the observed number of species (*p* < 0.05, Permutation *t* test), Chao1 (*p* < 0.05, Permutation *t*-test), Shannon (*p* < 0.0001, Permutation *t*-test), and Simpson (*p* < 0.0001, Permutation *t*-test), than horse-mice (Figures 1C,D; Supplementary Tables S1–S3). PCoA of Bray-Curtis distances indicated an apparent clustering by donor community and less by colonization time in the gut ([PERMANOVA] *F*-value: 57.165; R-squared: 0.37568; *value of p* < 0.001) (Figures 1E,F), which was supported by the PCoA of Jaccard distance (Supplementary Figure S2). Moreover, the UPGMA tree of weighted UniFrac distances of these samples showed two clear clusters by donor community (Supplementary Figure S3). Random forest analysis revealed that 17 ASVs played an important role in the significant differences between panda-mice and horse-mice (Figure 1G). Among these 17 ASVs, 9 ASVs had significantly higher abundance in horse-mice than panda-mice and 8 ASVs were significantly enriched in panda-mice (Figure 1G).

### The time points of stable gut microbiota composition

The number of observed species decreased sharply from gavage to the fourth day of colonization for both horse-mice (from 657 to 235, *p* < 0.05, Permutation *t*-test) and panda-mice (from 254 to 183) (Figure 1D; Supplementary Table S3). The Shannon diversity index of horse-mice decreased sharply (from 6.402 to 4.580, *p* < 0.01, Permutation *t*-test) whereas that of panda-mice increased from gavage during colonization of GF mice (from 2.038 to 4.084) (Figure 1C; Supplementary Table S2). So, an unstable stage of gut microbiome existed at the time when fecal microbiota was transplanted into GF mice, which is consistent with Seedorf et al. (2014).

**Figure 2:**
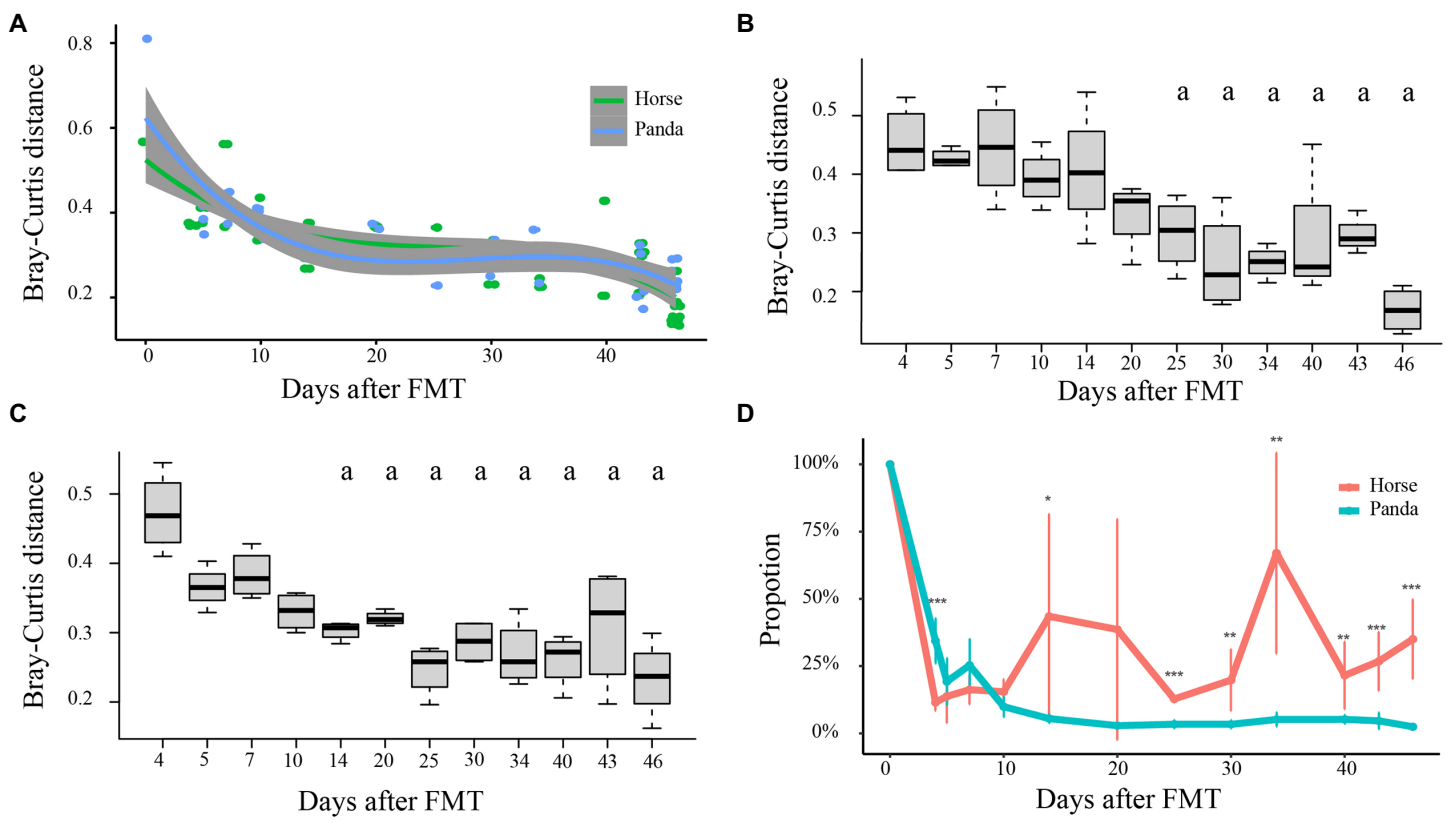
The time points of stabilization of the mice gut microbiota after FMT. Bray-Curtis distances of gut microbiota between samples taken at each time point after FMT and the median of corresponding donor samples at the last time point are shown in **(A)**. The statistical analysis of the Bray-Curtis distances of horse-mice between each time point and the median at the last time point after FMT is shown in **(B)** and that of panda-mice in **(C)**. Letter “a” in panels **(B)** and **(C)** indicated no significant difference with the last time point. Significance test for panels **(B)** and **(C)** was performed by applying the One-way ANOVA with post-hoc Tukey’s HSD (Honestly Significant Difference) by “glht” function in “multcomp” package. *t*-test was used for panel **(D)** (* *p* < 0.05, ** *p* < 0.01, *** *p* < 0.001). The results of source tracking analysis are shown in **(D)** and the proportion of the microbial community composition from the original horse or panda feces changed with days after the original microbial community colonized into corresponding horse-mice or panda-mice. Only at the 5^th^, 7^th^, 10^th^ or 20^th^ day, the retention proportions of the original donor microbial community composition were not significant between horse-mice and panda-mice.

Figure 2 shows the Bray-Curtis distances between every sample of horse-mice or panda-mice and the mean values for the last sampling time point (46 days after FMT) of corresponding horse-mice or panda-mice, respectively. The distances decreased sharply for both horse-mice and panda-mice, then the slopes of curves of the distances became gentle (Figure 2A), which showed that the gut microbiota began to stabilize. It was found that the initial time point was the 25^th^ day for horse-mice and the 14^th^ day for panda-mice when their Bray-Curtis distances showed no significant differences with those of the 46^th^ day after FMT (*p* > 0.05, One-way ANOVA) (Figures 2B,C). In other words, the gut microbiota stayed stable after 25 days after the fecal microbiota of horse was transplanted into GF mice and remained stable after 14 days for giant pandas, which was consistent with that of Huang et al. (2022).

The time points were supported also by UniFrac distances and source tracking analysis. The UPGMA tree of weighted UniFrac distances showed that the temporal pattern of assembly was consistent within donors of mice and most of the samples from more than 14 days after FMT of panda-mice clustered together and those from more than 25 days after FMT of horse-mice clustered together, too (Supplementary Figure S3). The UPGMA tree of unweighted UniFrac distances indicated that at the 43^rd^ day and 46^th^ day after FMT that the panda-mice or horse-mice microbiota clustered with their corresponding donors (Supplementary Figure S4). This was similar to the results from the PCoA where all different types of transplanted communities assembled within recipient GF mice over the course of more than 30 days (Figures 1E,F). The source tracking analysis showed that the proportion of the donor panda microbial community was approximately 4.0% with no significant variance (*p* > 0.05, One-way ANOVA) in panda-mice 14 days after FMT and that of the donor horse stayed relatively stable at about 28.3% in horse-mice at 40 days after FMT (Figure 2D).

Thus, although the time point when the gut microbiota in FMT mice turned stable was different for giant pandas and horses, they both suggest a possible “shock” period immediately after transplantation of the fecal microbiota, followed by adaptation and a stable state and the 43^rd^ day after transplantation into mouse would keep stable for giant panda and horse, a similar dynamic change of human fecal microbiota transplantation into GF mice was described recently (Li et al., 2021).

### The change of fecal bacterial communities from gavage to colonization

To further investigate the changes in specific taxa throughout time, we compared the relative abundance of the gut microbiota at the phylum and genus level following the methods of Li et al. (2021). We found that Bacteroidetes (52%), Firmicutes (20%), and Actinobacteria (0.7%) stayed relatively stable in donor horse and horse-mice and the relative abundance of the three phyla changed no more than twofold (Figure 1B). The relative abundance of Firmicutes increased more than twofold from donor panda (8%) compared to panda-mice (23%) when transplantation microbiota was stable. Donor pandas (80%) and horses (10%) harbored higher proportions of Proteobacteria, which then decreased more than twofold from the corresponding donor to FMT mice (16% for panda-mice and 4% for horse-mice; Figure 1B). Interestingly, although a large difference between the relative abundance of Bacteroidetes from donor pandas (12%) and from horses (40%) was observed, the relative abundance of Bacteroidetes showed no significant difference between panda-mouse and horse-mouse (*p* > 0.05, *t*-test; Figure 1B). At the family level, the abundance of Bacteroidaceae from both donors of giant pandas (from 7.48 to 42.7%) and horses (from 18.08 to 36.5%) increased, whereas the abundance of Enterobacteriaceae decreased significantly from 75.86% in donor pandas to about 10% in panda-mice (Supplementary Table S4).

After removing genera with relative abundances of less than 0.1, 67.9% (36/53) of the genera in horse–mice pairs and 85.7% (18/21) of the genera in panda–mice pairs were significantly changed with at least two-fold difference between donor and the median of gut microbiota of the corresponding mice at 43 and 46 days after FMT (Supplementary Table S4). The panda-mice and horse-mice pairs shared 9 genera and all of which significantly changed in the two pairs (Supplementary Table S4). Both pairs shared *Lachnospiraceae_NK4A136_group* and *Escherichia-Shigella* which changed significantly only in the panda-mice pair (Supplementary Table S4). In addition, Venn diagrams showed 121 shared ASVs across all time points for panda-mice, and 150 shared ASVs across all time points for horse-mice (Supplementary Figure S5). In addition, 7 ASVs from the donor giant pandas and 50 ASVs in donor horses never colonized the GF mice (Supplementary Figure S5). These results indicated that there are distinct host species preferences among giant panda, horse, and mouse microbiota.

Linear discriminant analysis effect size (LEfSe) analysis was used to identify bacterial taxa significantly contributing to the differences observed among time points after FMT for panda-mice (Supplementary Figure S6) or horse-mice (Supplementary Figure S7). Mice inoculated with the giant panda fecal microbial community showed higher levels of *Proteobacteria* and of *Firmicutes* at the 4^th^ (17%) and the 34^th^ day (32%) after FMT, respectively (Supplementary Figure S6). Mice inoculated with the horse microbiota had a higher relative abundance of Proteobacteria (8%) and Fusobacteria (3%) at the 34^th^ day after FMT (Supplementary Figure S7). In addition, the relative abundance of Proteobacteria (16%) in panda-mice remained stable for more than 10 days after FMT (Figure 1B; Supplementary Figures S1, S6).

The LEfSe analysis was also used to identify bacterial taxa significantly contributing to the differences observed between panda-mice and horse-mice when the corresponding microbiota showed a stable composition (around 43 and 46 days after FMT; Supplementary Figure S8). Animals colonized with the panda fecal microbial community showed higher levels of Firmicutes (26% for panda-mice and 20% for horse-mice) and Proteobacteria (17% for panda-mice and 5% for horse-mice), whereas mice colonized with the horse community had a higher relative abundance of Fusobacteria (0.07% for panda-mice and 2% for horse-mice), and Verrucomicrobia (0.5% for panda-mice and 20% for horse-mice; Supplementary Figure S8). At the genus level, panda microbiota-colonized animals showed a higher relative abundance of 12 genera; mice harboring the horse microbiota showed increased levels of 27 genera (Supplementary Figure S8).

The comparison of the relative abundance for each genus from the all the original donor’s microbiota and from all the GF mice in the last day showed that 169 of 534 genus were increased by the end of this study while 168 genera decreased. In the panda-mice, the relative abundance of both of the “increased group” and “decreased group” in the last day were significantly different to the original microbiota. The mostly increased genus was *Bacteroides* (increased 24.3%), and the most decreased genus was *Escherichia-Shigella* (decreased 36.4%). Other genera did not change more than 10% compared to the original microbiota. The relative abundance of the “increased group” of genera changed from averagely 14.2 to 58.4% (*p* = 0.0013, paired *t*-test) while the “decreased group” was from 84.4 to 32.0% (*p* = 0.0014, paired *t*-test) in the panda-mice. However, in the horse-mice, the “increased group” of genera slightly changed from 69.5 to 84.3% (*p* = 0.0023 paired *t*-test) while the “deceased group” did not change significantly (averagely from 8.67 to 8.66%, *p* = 0.99, paired *t*-test; Supplementary Figure S9).

Source tracking analysis showed that the proportion of the original panda microbial community decreased in panda-mice at the genus level (Figure 2D). The original panda microbial community composition reached – on average – 34.4% at the 4^th^ day, 19.2% at the 5^th^ day, 25.3% at the 7^th^ day, 10% at the 10^th^ day, then stayed at about 4.1% after the 14^th^ day after FMT. In contrast, a different pattern was observed in horse-mice. The original horse microbial community reached – on average - 11.5% at the 4^th^ day, 13.7% at the 5^th^ day, 16.2% at the 7^th^ day, 15.5% at the 10^th^ day, then increased to 33.5% on average after the 14^th^ day after FMT. At the 4^th^ day after FMT, the proportion of original panda microbial community in panda-mice was significantly higher than that of original horse microbial community in horse-mice (Figure 2D). After 25^th^ day of FMT, when the transplantation microbial community into mice remained relatively stable for both panda-mice and horse-mice, the proportion of panda donor microbial community in panda-mice was significantly lower than that of horse donor microbial community in horse-mice (Figure 2D).

### Correlation analysis between microbiota composition and metabolic profiles

Serum samples (*n* = 16, 8 for horse-mice and 8 for panda-mice) from the last time point (46 days after FMT) were used to analyze metabolites using liquid chromatography-mass spectroscopy (LC–MS) analysis and a total of 3,076 compounds was quantified (Supplementary Table S5). Out of the 3,076 compounds, 150 were annotated by the HMDB (Human Metabolome Database) and 549 by KEGG (Kyoto Encyclopedia of Genes and Genomes). These compounds were significantly enriched in two KEGG pathways, including the Arginine and Proline metabolism (map00330) and the Glycine, Serine, and Threonine metabolism (map00260) (Supplementary Table S5).

**Figure 3:**
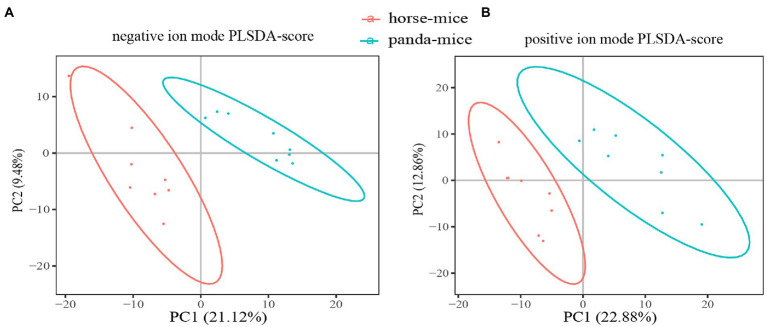
The multivariate statistical analysis of serum metabolites based on LC–MS in positive and negative ion mode. PLS-DA score plots of the serum metabolic profile between panda-mice and horse-mice in negative **(A)** and positive **(B)** ion mode were shown in here.

Principal component analysis (PCA) and partial least squares discrimination analysis (PLS-DA) both showed a significant separation of clusters between panda-mice and horse-mice, which was consistent with heatmap analysis revealing substantial alteration of metabolites (Figure 3; Supplementary Figure S8). Based on VIP (variable importance in the projection) > 1.0, FC (fold change) > 1.2 or < 0.833, and *p*-value <0.05 (*t*-test), a total of 239 serum metabolites were identified to be significantly different between panda-mice and horse-mice. Among those, 161 metabolites were significantly increased and 78 metabolites significantly decreased in horse-mice versus panda-mice (Supplementary Figure S11; Supplementary Table S5). Moreover, antibiotic metabolites were significantly down-regulated in panda-mice compared with horse-mice. In addition, hierarchical cluster analysis of these differential metabolites showed one sample, H1744 of horse-mice, did not cluster with other samples of horse-mouse and the reason was unknown (Supplementary Figure S11; Supplementary Table S5).

To identify bacterial taxa associated with metabolites, we calculated correlation matrixes based on Spearman correlation coefficients and clear correlations were indicated with *R* > 0.5 or *R* < −0.5 and *p* < 0.05. The 9 upregulated metabolites in panda-mice were positively correlated with [*Eubacterium*]_*xylanophilum_group*, which also negatively correlated with 25 metabolites involved in the biosynthesis of unsaturated fatty acids and metabolism of glycine, serine and threonine (Supplementary Figure S12). Three serum metabolites were positively correlated with the myxobacterium *Sandaracinus* which was negatively correlated with 10 metabolites (Supplementary Figure S12). The down-regulated metabolites in panda-mice, such as Tiglic acid and Sarcosine, were positively correlated with *Oscillibacter* and *Holdemania* (Supplementary Figure S12). Tiglic acid is an unsaturated short-chain fatty acid that can modify histones to achieve epigenetic regulation (Liao et al., 2020).

### The comparison between high and low retention proportions of giant panda gut microbiota in GF mice

We re-analyzed the 16 giant panda donor microbiotas that were inoculated into GF mice in the study by Huang et al. (2022) who also used FMT of Panda microbiota into GF mice, but is different from our study by focusing on growth features due to dietary differences based on different feeding seasons when giant pandas were feed either on bamboo shoots or on bamboo leaves. The results showed that some samples at day 0 did not cluster with panda-mice came from the paper of Huang et al. (2022) (Supplementary Figure S13), which was consistent with our above results.

**Figure 4:**
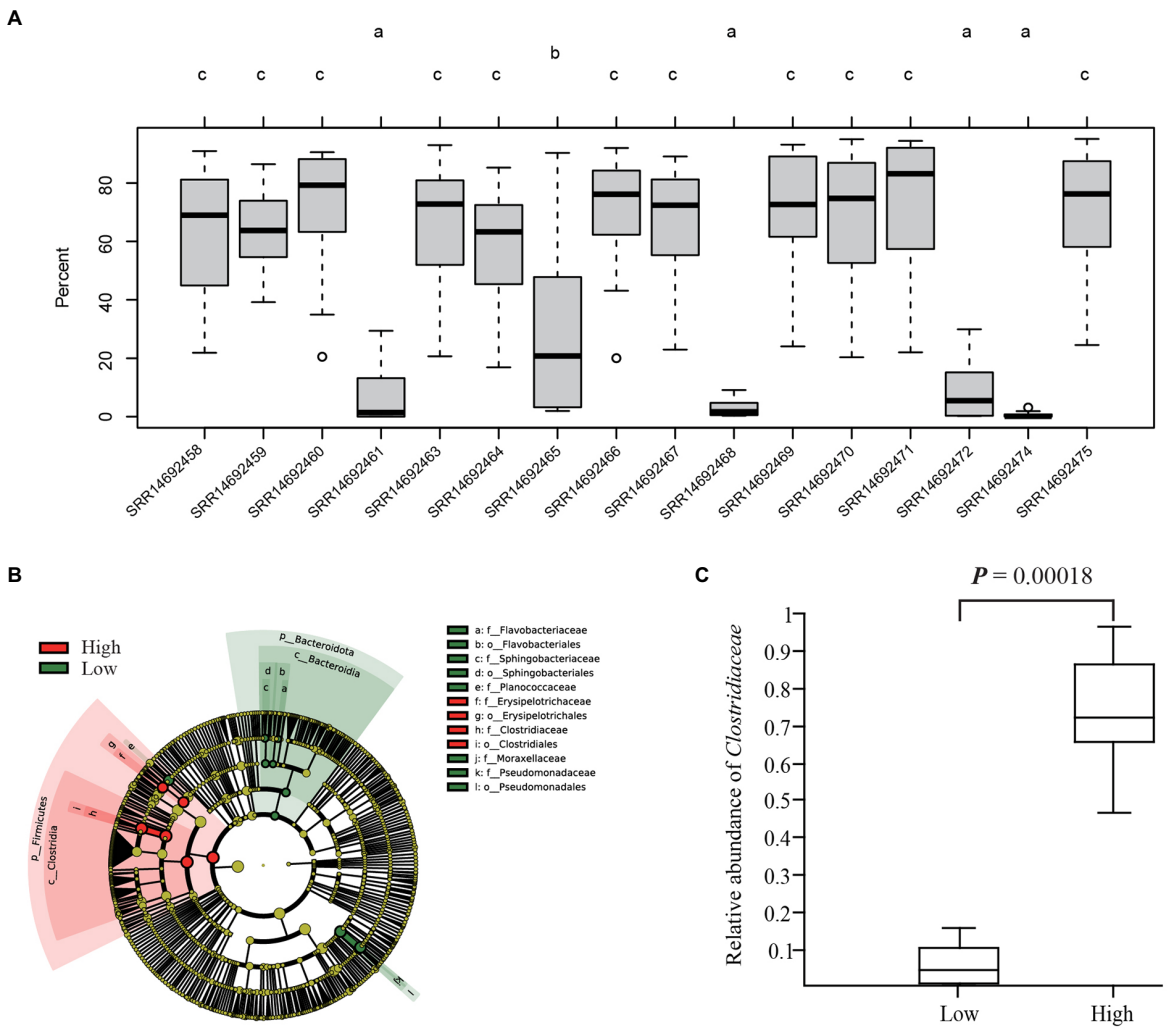
Differential abundance analyzes among the 16 giant panda donor microbiota from Huang et al. (2022). The comparisons of the proportions of 16 original giant panda fecal microbiotas in GF mice on 14^th^ or 21^st^ day after FMT with source tracking analysis indicated that the differences among 14^th^ or 21^st^ day were same (Supplementary Figure S12) and only results for the 21^st^ day are shown in **(A)**. Four fecal microbiotas had proportions lower than 25% (indicated by the letter a) and the proportions of the other 11 original fecal microbiotas were significantly higher (indicated by the letter c). Different letters indicate significant differences (*p*-value < 0.05; One-way ANOVA with post-hoc Tukey’s HSD test). Thus, the four samples (letter with a) were indicated with low percent group and the 11 samples (letter with c) with high percent group. The results of LEfSe analysis between the low and high percent groups is shown in **(B)**. Here *Clostridiaceae* showed significant difference between low and high groups. The relative abundance analysis of *Clostridiaceae* of 16 giant panda donor microbiota (including the 15 samples from panel **(A)** and our 1 sample in this study) between the low and high groups is shown in **(C)** (*t*-test).

Source tracking analysis of the 16 giant panda fecal microbiotas showed that all proportions of the original panda donor microbiotas were decreased in GF mice, especially for four samples that only contained less than 25% of the donor microbiotas at the last two time points (Supplementary Figure S14). Huang et al. (2022) found that the gut microbiota from giant pandas was already stably established 14 days after FMT, which was consistent with our results. Thus, we compared the differences of retention proportions in GF mice among the 16 donor microbiota at the 14^th^ or 21^st^ day after FMT and found that the 16 microbiota were divided into three groups, which included low and high percent groups (Figure 4A; Supplementary Figure S14). The difference of retention proportions between groups low (3.4% ± 1.4%) and high (67.5% ± 1.3%) was significant (*p*-value <0.01, *t*-test) (Figure 4A). The LEfSe analysis of the microbiota difference between the low and high groups found that five bacterial families (*Pseudomonadaceae*, *Planococcaceae*, *Flavobacteriaceae*, *Moraxellaceae* and *Sphingobacteriaceae*) were highly abundant in group low percent while two families (*Erysipelotrichaceae* and *Clostridiaceae*) were highly abundant in group high percent (Figure 4B). The relative abundances of the 7 families from the LEfSe analysis were used to check correlations with the mean proportions of the original microbiotas in GF mice at the 21^st^ day and we found that the Pearson correlation index (R) of *Clostridiaceae* was significant (*p* = 7.81E-07, *t*-test) and highest (*R* = 0.90141) among the 7 families (Supplementary Table S6). A significant difference of the relative abundance of *Clostridiaceae* was found between group high (73.6% ± 2.0%) and group low (6.1% ± 0.4%; *p* = 0.00018, *t*-test; Figure 4C).

## Discussion

In this study, we first characterized the colonization of the mouse gut by transplanting the gut microbiota obtained from giant pandas and horses into GF mice. We subsequently compared the differences in reshaping the gut microbiota structure of the GF mice receiving either the giant panda or the horse microbiota. In addition, we also evaluated the effects of the different FMTs on the intestinal microbiota structure and serum metabolites for the GF mice.

### Germ–free mice may not be well–suited to analyze the function of the gut microbiota of some giant pandas

FMT of human fecal samples into GF mice is a useful tool to analyze causal relationships between the gut microbiota and human phenotypes (Matson et al., 2018). In this study, we aimed to identify giant panda gut microbiota changes after transplantation into GF mice to determine whether GF mice could be used as a model to analyze the function of giant panda gut bacteria. The results showed transplantation of fecal microbial communities from giant panda feces into GF mice can only re-establish a portion of the donor microbiota and a bigger difference in panda-mice than horse-mice was observed. We found that 79.2% (42/53) of horse gut microbes could be re-established in GF mice at the genus level, among which 59.5% were significantly changed after FMT following the methods of Li et al. (2021). Strikingly, at the genus level, only 71.4% (15/21) of giant panda gut microbes could be re-established in GF mice, among which 86.7% underwent significant changes (Supplementary Table S4). Li et al. (2021) found that 60% of the human gut microbes could be re-established in GF mice at the genus level, among which only 38.9% underwent significant changes. Thus, only about 9% [71.4% *(1–86.7%)] of giant panda donor microbiota can be re-established in GF mice with no significant change compared with 32% [79.2% *(1–59.5%)] for horse donor microbiota and 37% [60% *(1–38.9%)] for human microbiota. These results showed that the gut microbiota of different donor species have different capabilities to colonizing GF mice. This was consistent with Contijoch et al. (2019) who found that the lion and red panda microbiota reached higher densities in the mouse than in their native hosts and that the elephant and ferret microbiotas colonized mice at densities comparable to those in their native hosts and all transferred microbiota were significantly less dense than mouse gut microbiota transplanted into GF Swiss Webster mice. Our results also showed that the gut bacteria of herbivorous horse were able to successfully colonize GF mice similar to members of the human gut microbiota.

Pairwise comparisons using Wilcoxon rank-sum tests of the Bray-Curtis distances among samples from the same days after FMT showed that the differences between samples on the 14^th^ day were biggest for the horse-mice and smallest for the panda-mice (Supplementary Figures S15A,B). The gut microbiota differences remained stable for more than 25 days after FMT for both panda-mice and horse-mice (Figures 2A–C). After that, only two pairwise comparisons for horse-mice (days 14–34, *p* < 0.001; 34–43, *p* < 0.05; Wilcoxon rank-sum test; Supplementary Figure S15A) and six pairwise comparisons for panda-mice (days 10–46, 14–34, 43–46, *p* < 0.05; 14–46, 25–46, 20–46, *p* < 0.001; Wilcoxon rank-sum test; Supplementary Figure S15B) were significant when the sample’s distance within same day more than 25 days after FMT were compared with other days, which indicated that the gut microbiota of panda-mice had a larger change range than that of the horse-mice. Moreover, the PCoA analysis showed that the donor panda microbiota did not cluster with panda-mice, whereas the donor horse microbiota did cluster with horse-mice (Figures 1E,F). The difference of Bray-Curtis distances between the panda donor microbiota and panda-mice microbiota (mean = 0.77669, SD = 0.00537) were significant larger than that between the horse donor microbiota and horse-mice microbiota (mean = 0.59177, SD = 0.001644; *p* = 0.0001046, Wilcoxon rank-sum test). Furthermore, by comparing the bacterial genera that changed by the end of this experiment in terms of the relative abundance, we found that this change can be mainly explained by the instability of the microbiota in panda-mice. Bacterial genera changed significantly in the panda-mice model compared to the mild-to-no changes in the horse-mice (Supplementary Figure S9). Thus, a larger gut microbiome variation between giant panda donors in this study and GF mice recipients than that between horse donor and GF mice recipients was revealed. However, the microbiome of GF mice after FMT still likes their donors (Supplementary Figure S13).

In order to find the reason for the considerable variation in the gut microbiota between giant panda donor microbiota in this study and GF mice recipients, we analyzed the 16 giant panda donor microbiotas used to inoculate GF mice in the study by Huang et al. (2022). We found that the most likely reason for the low retention rate of the panda donor microbiota in GF mice was low relative abundance of *Clostridiaceae* in the panda donor microbiota (Figure 4), which also supported the results of Huang et al. (2022). However, we found the relative abundance of *Clostridiaceae* in the horse donor microbiota was also low (1.5%) but with a high retention proportion in GF mice (30.9% ± 5.1%). These differences may be due to the microbiota having adapted to the intestinal architecture and fermentation of its native host. Mice have a large cecum, which is an important site for fermentation (Hugenholtz and de Vos, 2018). The giant panda’s cecum is absent and its large intestine lacks fermentation capacity (Hirayama et al., 1989), and the degradation capacity of cellulose in bamboo was weak (Zhang et al., 2018). The microbes in the horse hindgut are primarily responsible for the fermentation of complex polysaccharides (Donaldson et al., 2016).

In addition, we only analyzed FMT of giant pandas into GF Balb/c mice. Zhou et al. (2019) found that mouse genotypes exerted different selective pressures on exogenous colonizers. Wos-Oxley et al. (2017) reported that the genetic background of the various recipient rodents (rats and mice) strongly influenced the nature of the populating human gut microbiota, determining each model’s biological suitability. These two aforementioned studies proved that members of the phylum Bacteroidetes were well established in all rodent models, mice enriched for phylotypes related to species of *Bacteroides*, which was consistent with our results that mice were well suited to establish members of the phylum Bacteroidetes of giant pandas and horses (Figure 1; Supplementary Table S4). Although the horse group microbiota had high variation at many time points and large overall fluctuation (Figure 2D), they remained stable at 40 days after FMT and the proportion of horse-mice microbial community was significantly larger than that of panda-mice at the time points when horse-mice and panda-mice showed stable microbiota, so our conclusions are reliable. The reason for the large fluctuation in the horse group may be that the donor horse gut microbiota needed more time than that of the panda to establish stable in GF mice recipients, which was also supported by Bray-Curtis and UniFrac distances (Figures 2B–D; Supplementary Figures S3, S4). Taken together, our results are consistent and support our hypothesis.

FMT has already indicated an enormous potential for the improvement of both the management and conservation of wildlife (Guo et al., 2020) and has recently garnered renewed interest in veterinary medicine (Mullen et al., 2018). It also plays an important role to reveal evolutionary adaptation of wild animals and to check the potential mechanism of detoxification of cyanide compounds by gut microbiomes of giant pandas found by Zhu et al. (2018). However, the stability of transplanted microbiota could be affected by disease processes, treatment practices and supplementation (Mullen et al., 2018). The results of our study indicated that the bacterial abundance of certain genera from microbiota of giant panda, such as *Bacteroides* and *Escherichia-Shigella* changed more obviously than other genera after being transplanted for 46 days, while the abundance of other genera were relatively stable. Therefore, although the re-established microbiota may function as predicted in the GF mice and can remain some characteristics, it is still necessary to consider the potential changes of the transplanted giant panda microbiota in researches with GF mice.

### A smaller effect on the microbiota from the diet than from the donor

Diet is one of the key factors affecting the composition of gut microbiomes (Bolnick et al., 2014; Aira et al., 2015). An earlier study showed that carnivores had the lowest diversity of gut microbiota, omnivores were in the middle, and herbivores had the highest diversity (Ley et al., 2008). Our results are consistent with this previous research showing that the intestinal microbial diversity of horses is higher than that of giant pandas.

In addition, host species also has been reported as one key factor shaping the gut microbiota (Nishida and Ochman, 2018; Knowles et al., 2019). The gut microbes form a complex ecosystem and depend on the internal environment of the host, and interact actively with their host (Perofsky et al., 2018). However, this hypothesis often fails to explain the convergence of the gut microbial communities when the host species share diet or habitat, although the host species are only distantly related (Groussin et al., 2017; Perofsky et al., 2018). Amato et al. (2019) found bidirectional interactions of host physiology and gut microbiota over evolutionary time ultimately dictated the host nutritional outcomes resulting from a given dietary strategy. Thus, it is difficult to assess the influencing factors on the gut microbial community in natural environments.

To compare the effect of diet and microbial donor, we transplanted the fecal microbiota of giant panda and horse into GF mice and assessed whether there were significant differences in the colonization patterns with mice kept under the same diet and environmental conditions. Mice colonized with the same donor preserved a core of common species that differentiated it from mice colonized with the other donor community (Supplementary Figure S5). PCoA plots showed that microbial community structures were dramatically different among the recipient mice (Figures 1E,F). This was consistent with PCA and PLS-DA of the blood metabolites that showed significant separation of clusters between panda-mice and horse-mice, although diet and environment were identical (Figure 3; Supplementary Figures S10, S11). These results suggested that related taxa from both communities respond differently to a given diet.

We also assessed whether the significant differences in the two phenotypes would decrease from day 0 to day 46 after FMT when exposed to the same diet. Our results also revealed that microbial community membership and structure showed significantly higher intra-individual variations between panda-mice and horse-mice than that within panda-mice or horse-mice (*p* < 0.05; PERMANOVA with Monte Carlo). The Bray-Curtis distances among same day after FMT between panda-mice and horse-mice decreased significantly from day 4 to day 20 after FMT, the distances at 4 and 5 days after FMT were significantly higher than at the 7^th^, 10^th^, 14^th^, and 20^th^ day after FMT (*p* < 0.01; Wilcoxon rank-sum test). The distances increased significantly from day 20 to day 46 after FMT. The last two time points (43^rd^ and 46^th^ day after FMT) revealed the highest distances (*p* < 0.001; Wilcoxon rank-sum test) (Supplementary Figure S15C). These results showed that the difference of gut microbiota between GF mice with different donor microbiota would be decreased at the first few days after FMT (Supplementary Figure S15C), which indicated that the same diet and environment did play an important role on gut microbiota (Ley et al., 2008; Bolnick et al., 2014), however, the difference would be increased when the microbiota became stable after FMT (Supplementary Figure S15C), so the same diet and environment did not lead to higher similarity between the microbiota of the different host donors and the microbiome of GF mice after FMT still likes their donors (Supplementary Figure S13). Our results were consistent with other researchers who found that the structure and function of the gut microbiota is much stronger than that of the dietary niche (Galbreath et al., 2009; Perofsky et al., 2018; Amato et al., 2019).

Altogether, these results underscore the importance of the donor microbiota and prove a higher importance of the gut microbiota than the diet.

## Data availability statement

The datasets presented in this study can be found in online repositoriess. The names of the repository/repositories can be found in Supplementary material. The 16S rRNA datasets are available in the NCBI Sequence Read Archive database (accession number PRJNA902528).

## Ethics statement

The animal study was reviewed and approved by the Institutional Animal Care and Use Committee (IACUC) at Chengdu Research Base of Giant Panda Breeding (2019015).

## Author contributions

WZ, JX, SX, and XF performed the data and statistical analysis and drafted the manuscript. WZ, JX, and BZ carried out the germ-free mouse experiment. SS-E helped to draft the manuscript. LijZ, HH, HW, JZ, ZZ, and LiaZ participated in the data and statistical analysis. JX participated in the design of the study. WZ, MJ, and RH conceived the study and participated in its design and coordination. All authors read and approved the final manuscript.

## Funding

This work was supported by Application Foundation of Science & Technology Department of Sichuan Province under Grant 2019YJ0637; Chengdu Research Base of Giant Panda Breeding under Grant CPB2016-02, 2020CPB-B13, and 2021CPB-B10; Chengdu Giant Panda Breeding Research Foundation under Grant CPF2017-11. Innovative Postgraduate Research Master Key Project for Southwest Minzu University under Grant CX2019SZ81; and the Program for Innovative Research Team of Chengdu Normal University under Grant CSCXTD2020A04.

## Conflict of interest

The authors declare that the research was conducted in the absence of any commercial or financial relationships that could be construed as a potential conflict of interest.

## Publisher’s note

All claims expressed in this article are solely those of the authors and do not necessarily represent those of their affiliated organizations, or those of the publisher, the editors and the reviewers. Any product that may be evaluated in this article, or claim that may be made by its manufacturer, is not guaranteed or endorsed by the publisher.

## Footnotes

^1^ https://qiime2.org/

^2^ http://huttenhower.org/galaxy/

^3^ http://www.ehbio.com/ImageGP/

^4^ http://xcmsonline.scripps.edu/

^5^ http://www.MetaboAnalyst.ca/

^6^ http://www.hmdb.ca/

^7^ http://www.kegg.com/

## Supplementary material

SUPPLEMENTARY FIGURE S1 The relative abundance of top 6 families (up) and more than 1% genus (down) for horse (horse) and giant panda (panda).

SUPPLEMENTARY FIGURE S2 The PCoA plot with PCo1 and PCo2 based on Jaccard distance of 16S rRNA gene. The sample name was shown in here. More details of the sample information were shown in Supplementary Table S1.

SUPPLEMENTARY FIGURE S3 The UPGA tree basing on the weighted UniFrac distances of all samples. The samples with bolded and italicized words represent samples that belong to >= 14 days group for horse.

SUPPLEMENTARY FIGURE S4 The UPGA tree basing on the unweighted UniFrac distances of all samples.

SUPPLEMENTARY FIGURE S5 The venn chart of observed species: Unique ASV for every group. Here, H indicates horse and P indicates giant panda.

SUPPLEMENTARY FIGURE S6 The comparison results of LEfSe analysis among days after FMT for panda-mice.

SUPPLEMENTARY FIGURE S7 The comparison results of LEfSe analysis among days after FMT for horse-mice.

SUPPLEMENTARY FIGURE S8 The comparison results of LEfSe analysis between panda-mice (P) and horse-mice (H). The results are based on the combined data with no less than 34 days after FMT for panda-mice or horse-mice.

SUPPLEMENTARY FIGURE S9 Changes of bacterial genera by time in the panda-mice and horse-mice. **(A)** the panda-mice; **(B)** the horse-mice. Average relative abundance with standard deviations was showed at each time point. The “increased group” and “decreased group” were genera that changed correspondingly in the last day compared to the original donor’s microbiota across all types of donors.

SUPPLEMENTARY FIGURE S10 Principle component analysis (PCA) of serum metabolites of the mouse in positive (up) and negative (down) ion mode. H indicates horse-mice and P indicates panda-mice.

SUPPLEMENTARY FIGURE S11 Hierarchical cluster analysis of identified differential metabolites between panda-mice and horse-mice. Here, the metabolites with negative and positive ion mode were clustered and shades of blue and red represent down-regulation or up-regulation, respectively.

SUPPLEMENTARY FIGURE S12 The network of Spearman correlations of metabolite and bacterial features based on the 16S rRNA gene sequencing results. The type of correlation is indicated. Only significant correlations (*p* < 0.01) exhibiting an R > 0.5 or R < –0.5 were considered.

SUPPLEMENTARY FIGURE S13 The PCoA plot with PCo1 and PCo2 based on Bray-Curtis distance of 16S rRNA gene. Panda_Huang showed the samples from Huang et al. (2022). The numbers in this plot showed the time points.

SUPPLEMENTARY FIGURE S14 The changes of average proportions of giant panda fecal microbiota in GF mice at 7th, 14th, and 21st day after FMT. The data came from the reference (Huang et al., 2022).

SUPPLEMENTARY FIGURE S15 Comparisons of pairwise Bray-Curtis distances of gut microbiota among GF mice for each time point. **(A)** The distance among horse-mice; **(B)** the distance among panda-mice; **(C)** the distance between horse-mice and panda-mice for corresponding time points. The lines and squares inside boxes represent the median and mean, respectively. Wilcoxon rank-sum test was used in here and **p* <0.05, ***p* <0.01, ****p* <0.001.

SUPPLEMENTARY TABLE S1 The information of samples in this study.

SUPPLEMENTARY TABLE S2 The statistical analysis of Shannon index between pairwise groups for giant panda (P) and horse (H) in Figure 1B. See Supplementary Table S1 for the group details.

SUPPLEMENTARY TABLE S3 The statistical analysis of observed species between pairwise groups for giant panda (P) and horse (H) in Figure 1C. See Supplementary Table S1 for the group details.

SUPPLEMENTARY TABLE S4 The change between day zero and the last two time points after FMT at genus level for panda and horse (abundance > 0.1%).

SUPPLEMENTARY TABLE S5 The metabolic data in this study.

SUPPLEMENTARY TABLE S6 The correlation index (R) and P value between the proportions in GF mice on the 21st day and the relative abundance of the families obtained from LEfSe analysis of the 16 giant panda donors of Huang et al. (2022). The proportions in GF mice were obtained from source tracking analysis with the 16 samples of Huang et al. (2022) and one sample in this study. More details can be found in the text.

## Abbreviations

GF: germ-free
PLS-DA: partial least squares discrimination analysis
FMT: fecal microbial transplantation
ASVs: Amplicon Sequencing Variants
PCoA: principal coordinates analysis
LEfSe: linear discriminant analysis effect size
LC–MS: liquid chromatography-mass spectroscopy
HMDB: Human Metabolome Database
KEGG: Kyoto Encyclopedia of Genes and Genomes
VIP: variable importance in the projection
FC: fold change
PCA: principal component analysis
OPLS-DA: orthogonal projection to latent structure-discriminant analysis
