# Supplementary figures and images for: Evaluating a potential model to analyze the function of the gut microbiota of the giant panda
Source: Front Microbiol. 2022 Dec 20;13:1086058. doi: 10.3389/fmicb.2022.1086058 (PMC9808404; doi:10.3389/fmicb.2022.1086058)

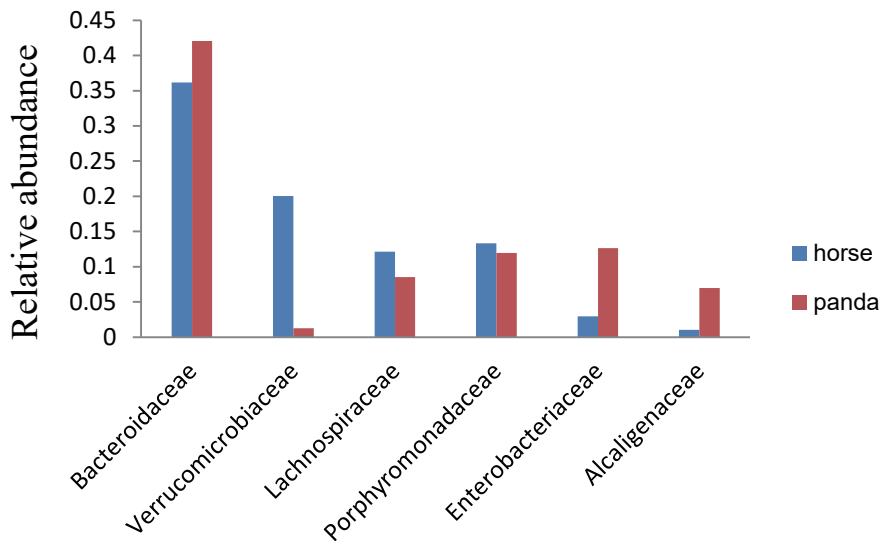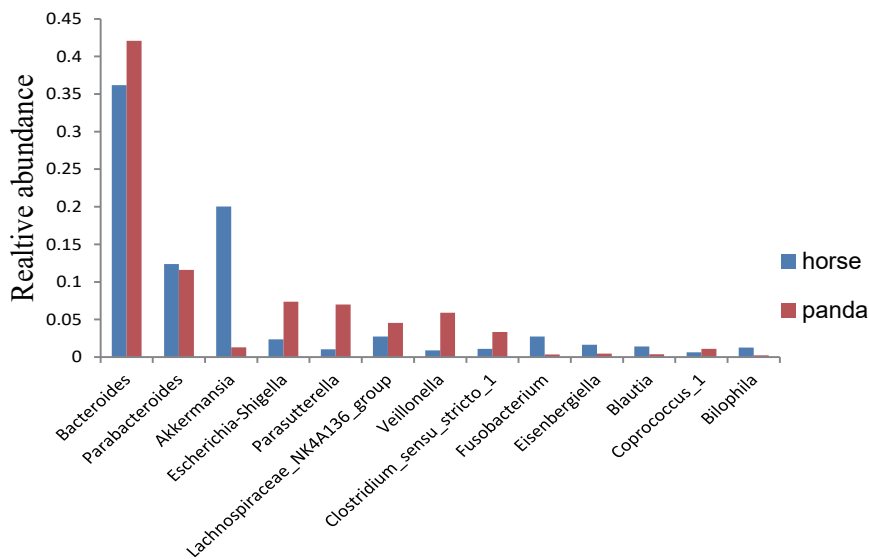

Supplement: SUPPLEMENTARY FIGURE S1 — The relative abundance of top 6 families (up) and more than 1% genus (down) for horse (horse) and giant panda (panda). [file Data_Sheet_1.PDF]

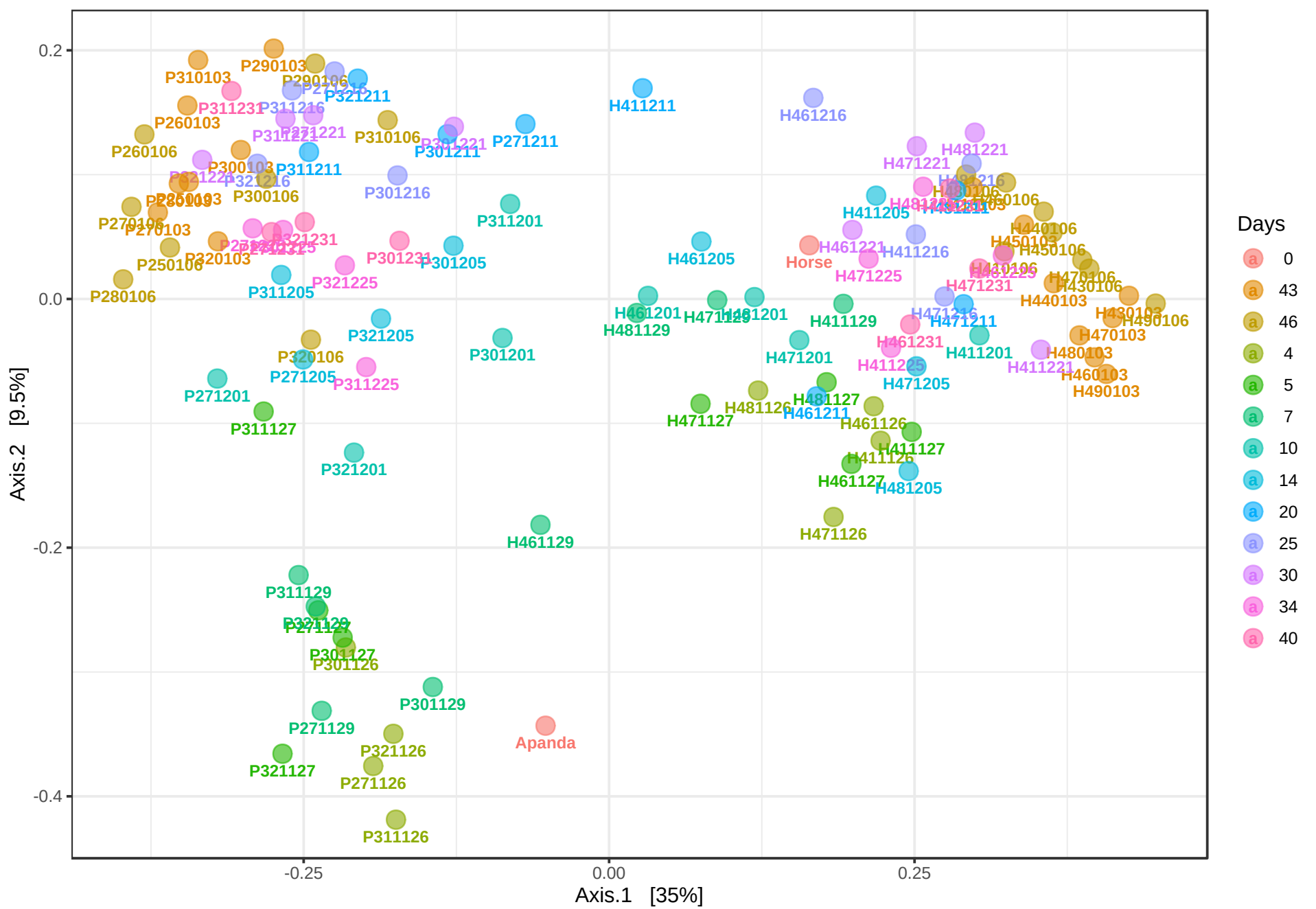

Supplement: SUPPLEMENTARY FIGURE S2 — The PCoA plot with PCo1 and PCo2 based on Jaccard distance of 16S rRNA gene. The sample name was shown in here. More details of the sample information were shown in Supplementary Table S1. [file Data_Sheet_2.PDF]

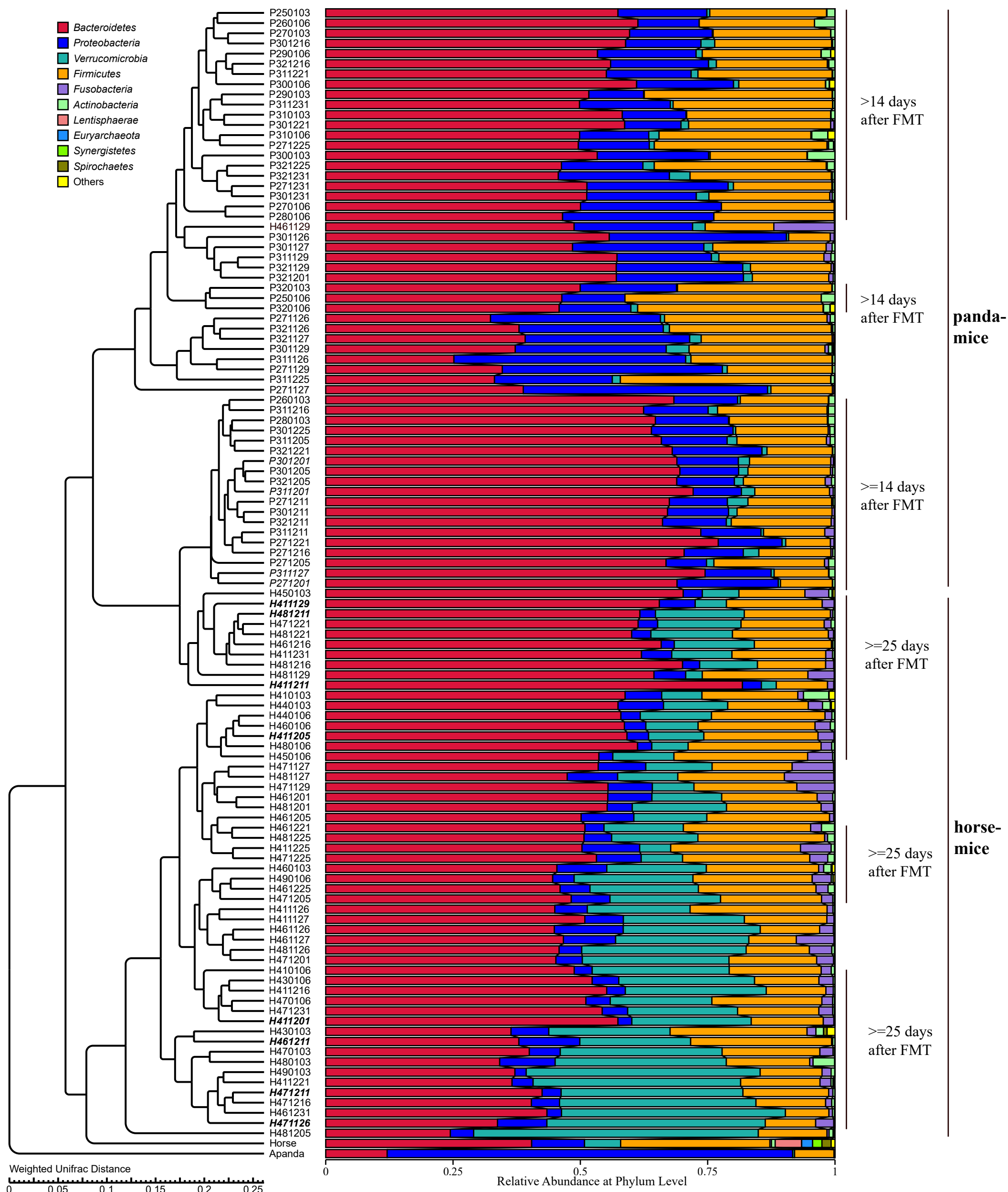

Supplement: SUPPLEMENTARY FIGURE S3 — The UPGA tree basing on the weighted UniFrac distances of all samples. The samples with bolded and italicized words represent samples that belong to >= 14 days group for horse. [file Data_Sheet_3.PDF]

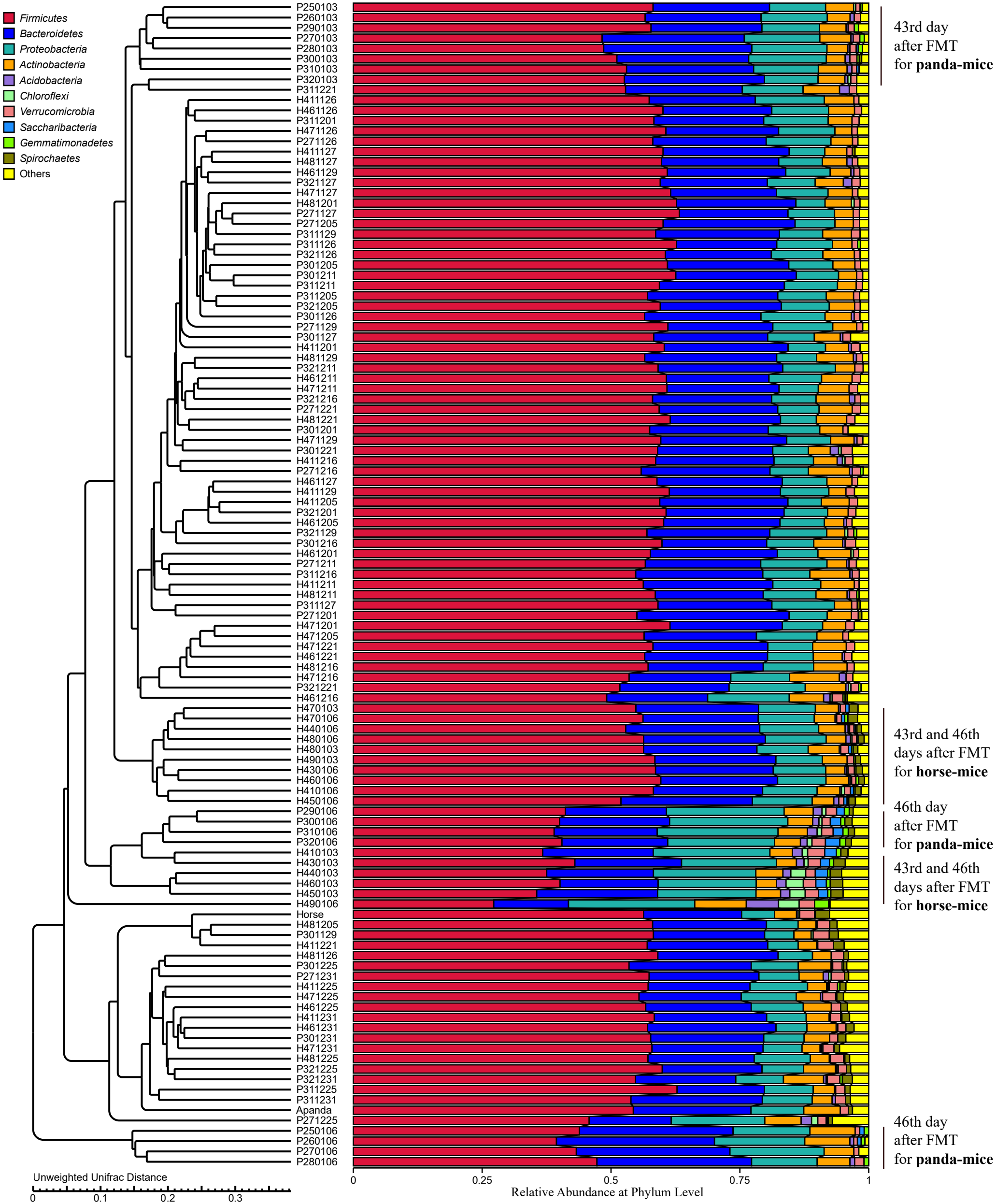

Supplement: SUPPLEMENTARY FIGURE S4 — The UPGA tree basing on the unweighted UniFrac distances of all samples. [file Data_Sheet_4.PDF]

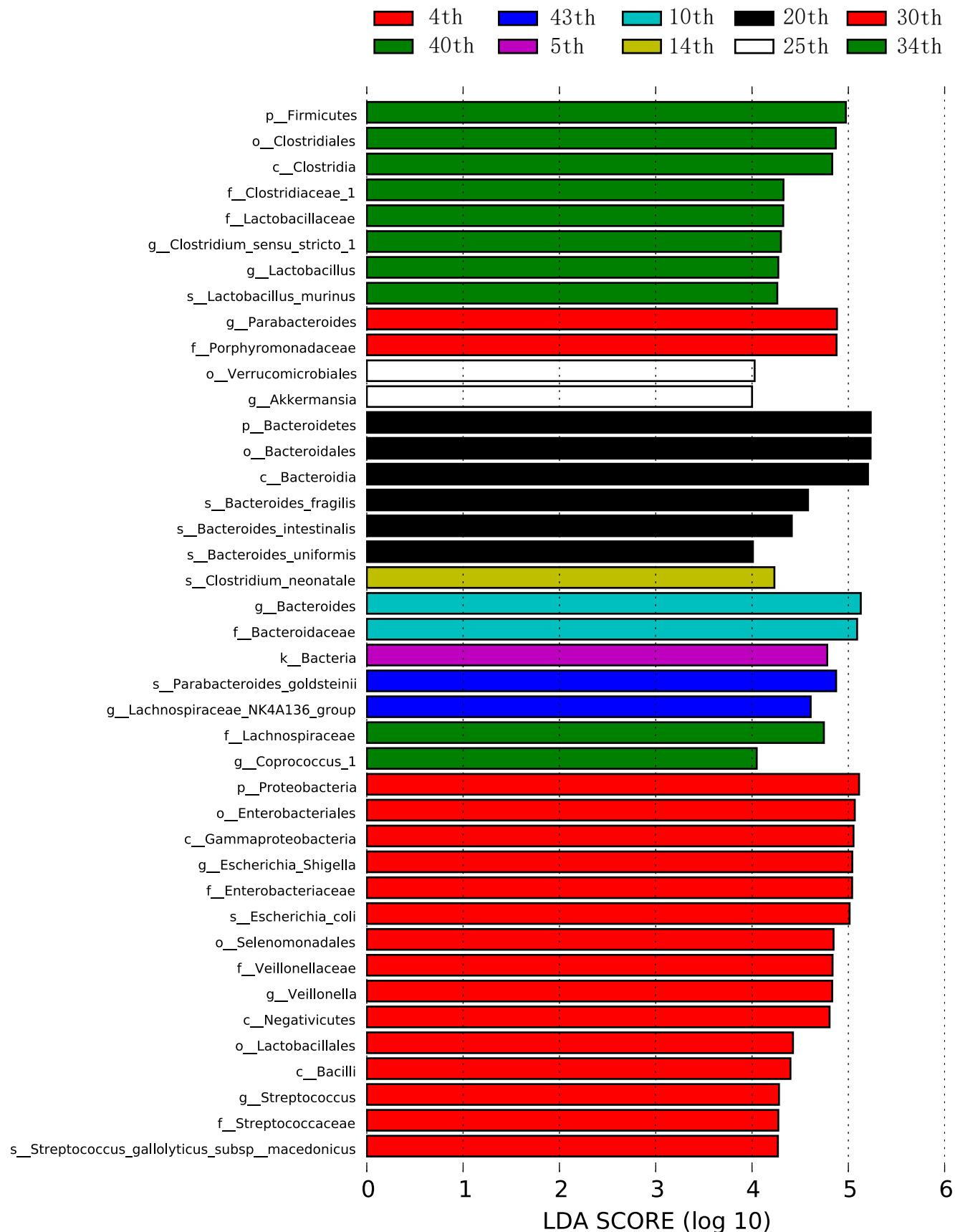

Supplement: SUPPLEMENTARY FIGURE S5 — The venn chart of observed species: Unique ASV for every group. Here, H indicates horse and P indicates giant panda. [file Data_Sheet_5.PDF]

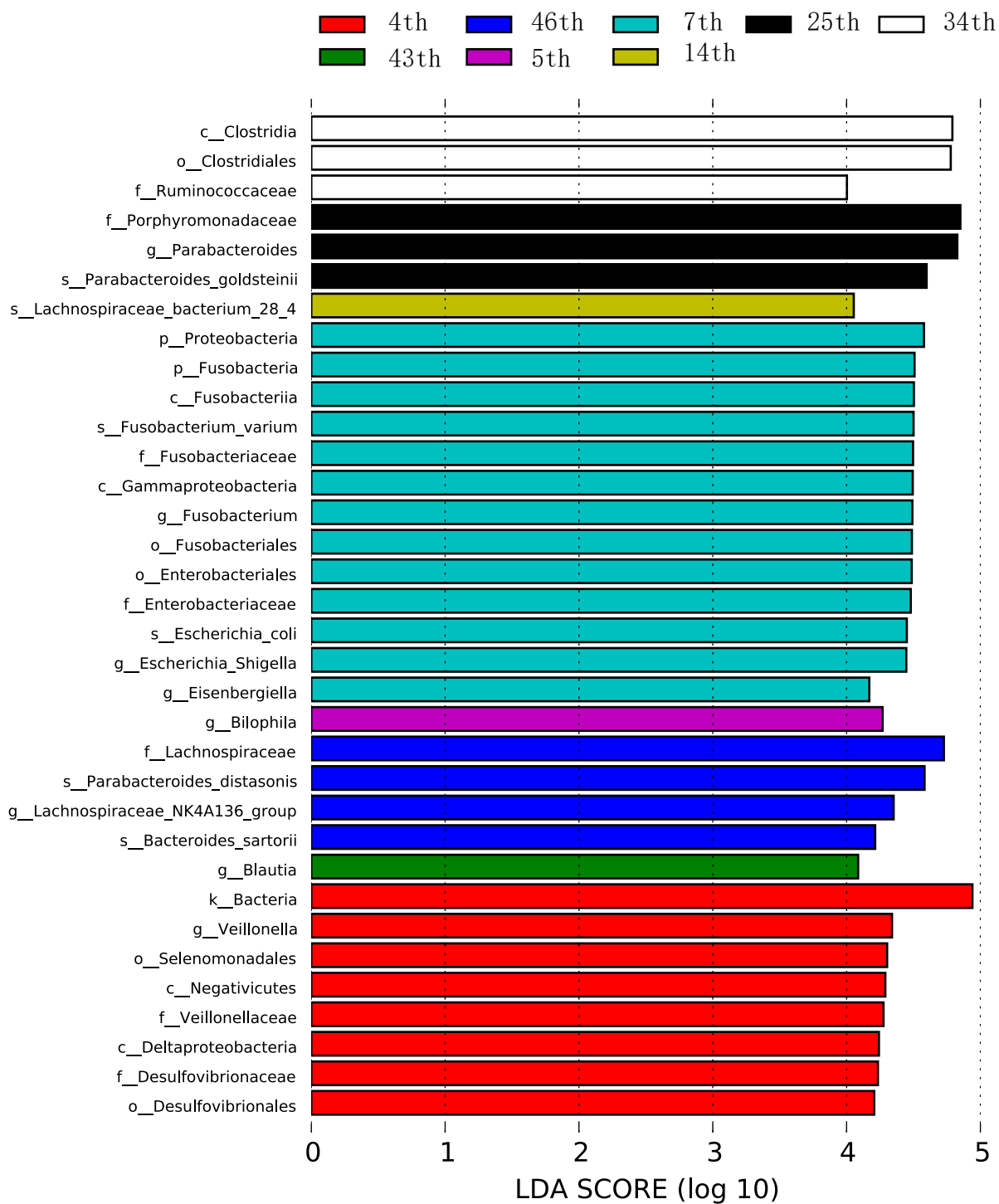

Supplement: SUPPLEMENTARY FIGURE S6 — The comparison results of LEfSe analysis among days after FMT for panda-mice. [file Data_Sheet_6.PDF]

**A**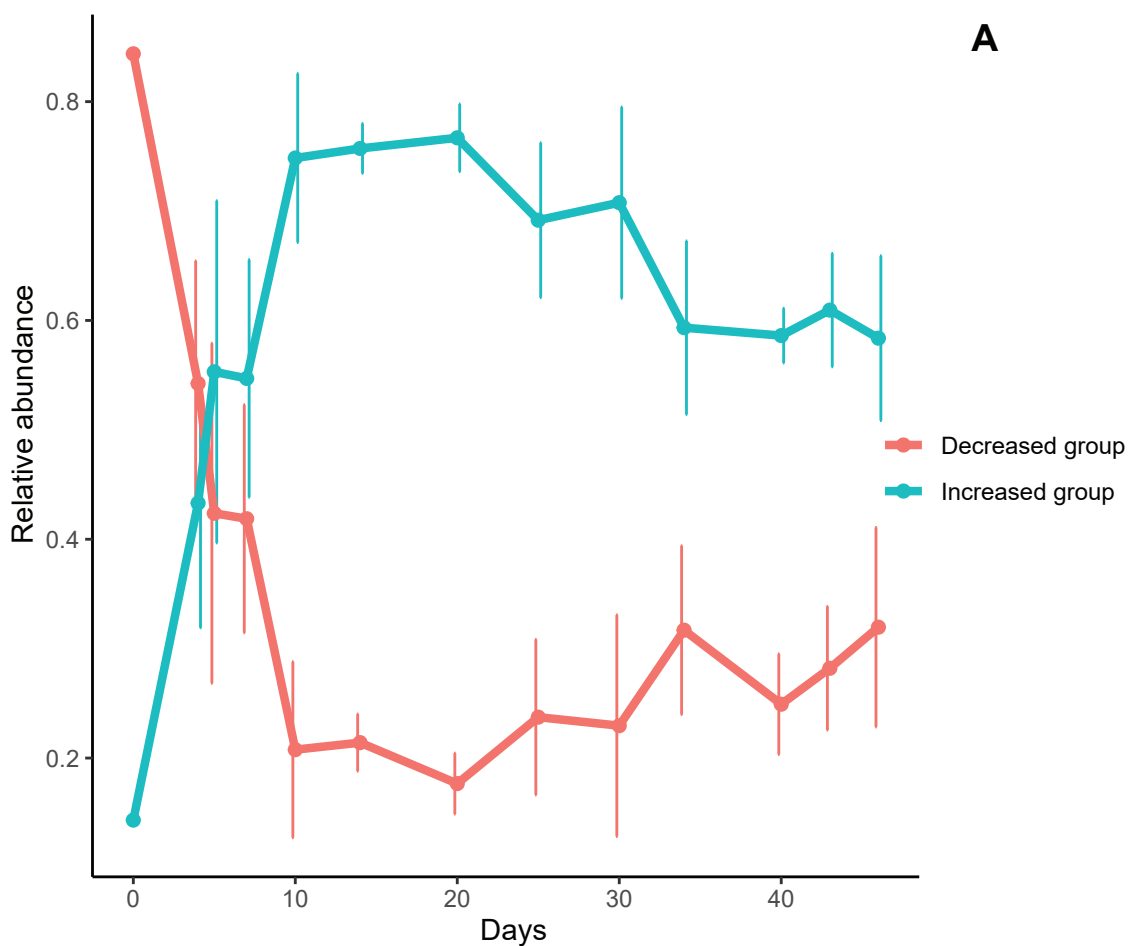**B**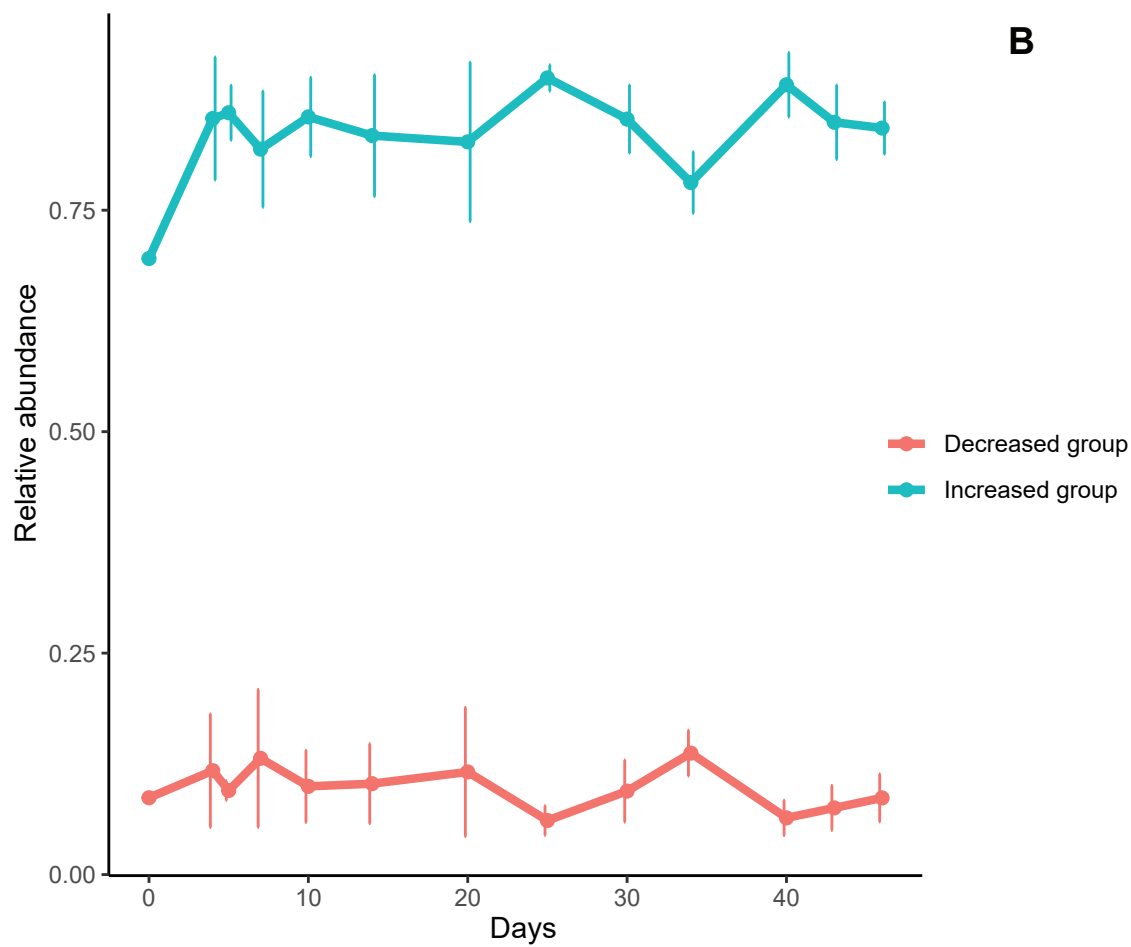

Supplement: SUPPLEMENTARY FIGURE S7 — The comparison results of LEfSe analysis among days after FMT for horse-mice. [file Data_Sheet_7.PDF]

• H • P

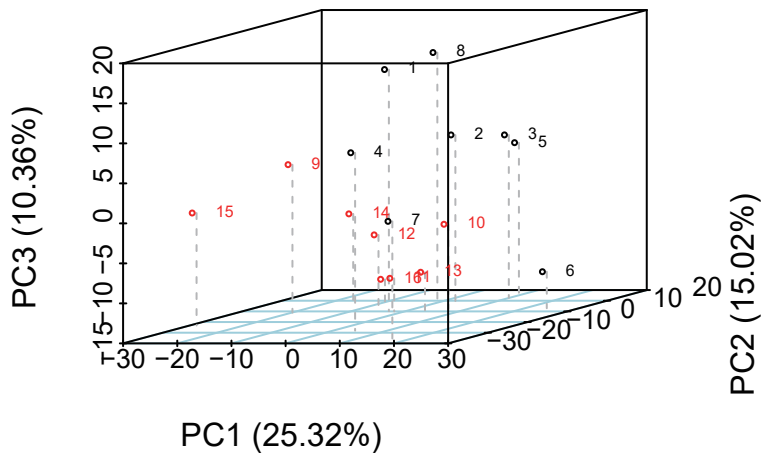

• H • P

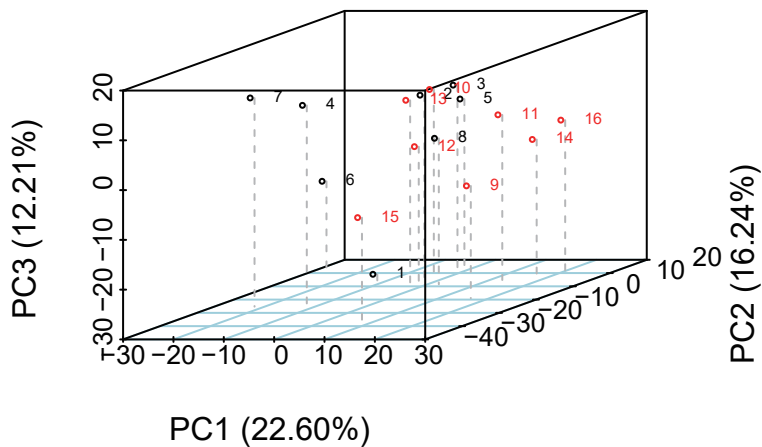

Supplement: SUPPLEMENTARY FIGURE S8 — The comparison results of LEfSe analysis between panda-mice (P) and horse-mice (H). The results are based on the combined data with no less than 34 days after FMT for panda-mice or horse-mice. [file Data_Sheet_8.PDF]

## Panda-mice vs. horse-mice (positive ion)

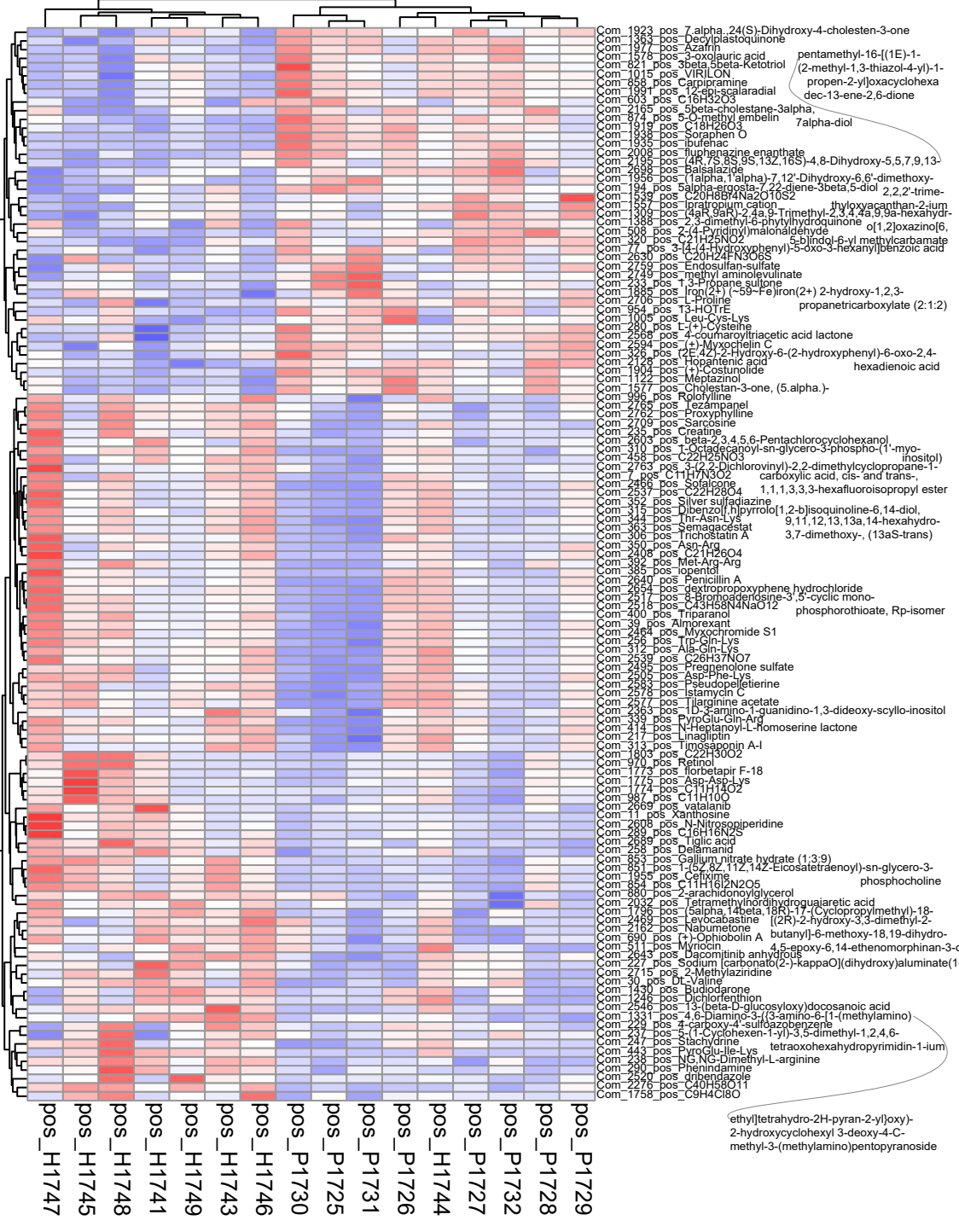

Supplement: SUPPLEMENTARY FIGURE S9 — Changes of bacterial genera by time in the panda-mice and horse-mice. (A) the panda-mice; (B) the horse-mice. Average relative abundance with standard deviations was showed at each time point. The “increased group” and “decreased group” were genera that changed correspondingly in the last day compared to the original donor’s microbiota across all types of donors. [file Data_Sheet_9.PDF]

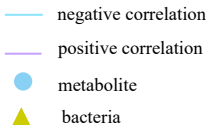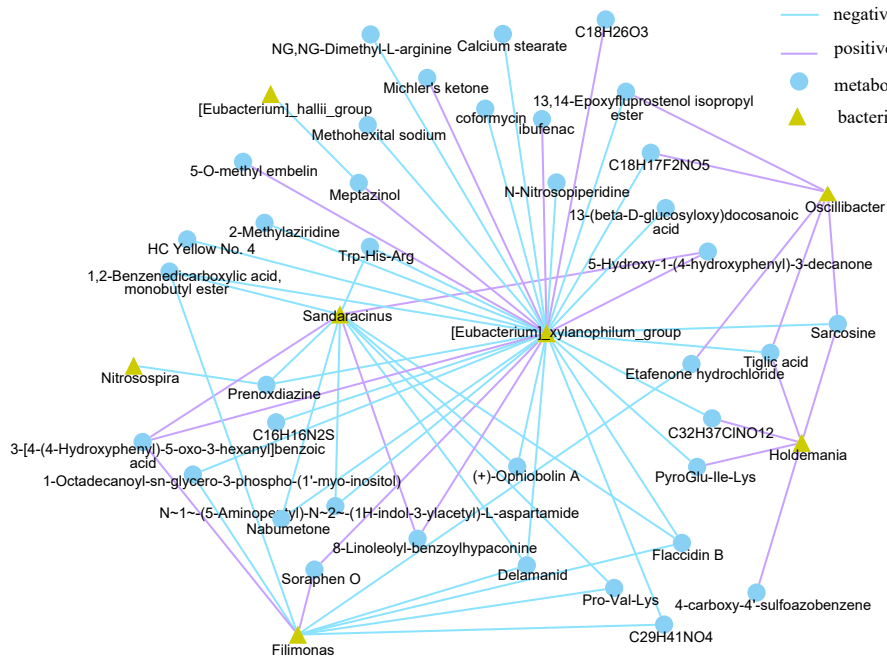

Supplement: SUPPLEMENTARY FIGURE S10 — Principle component analysis (PCA) of serum metabolites of the mouse in positive (up) and negative (down) ion mode. H indicates horse-mice and P indicates panda-mice. [file Data_Sheet_10.PDF]

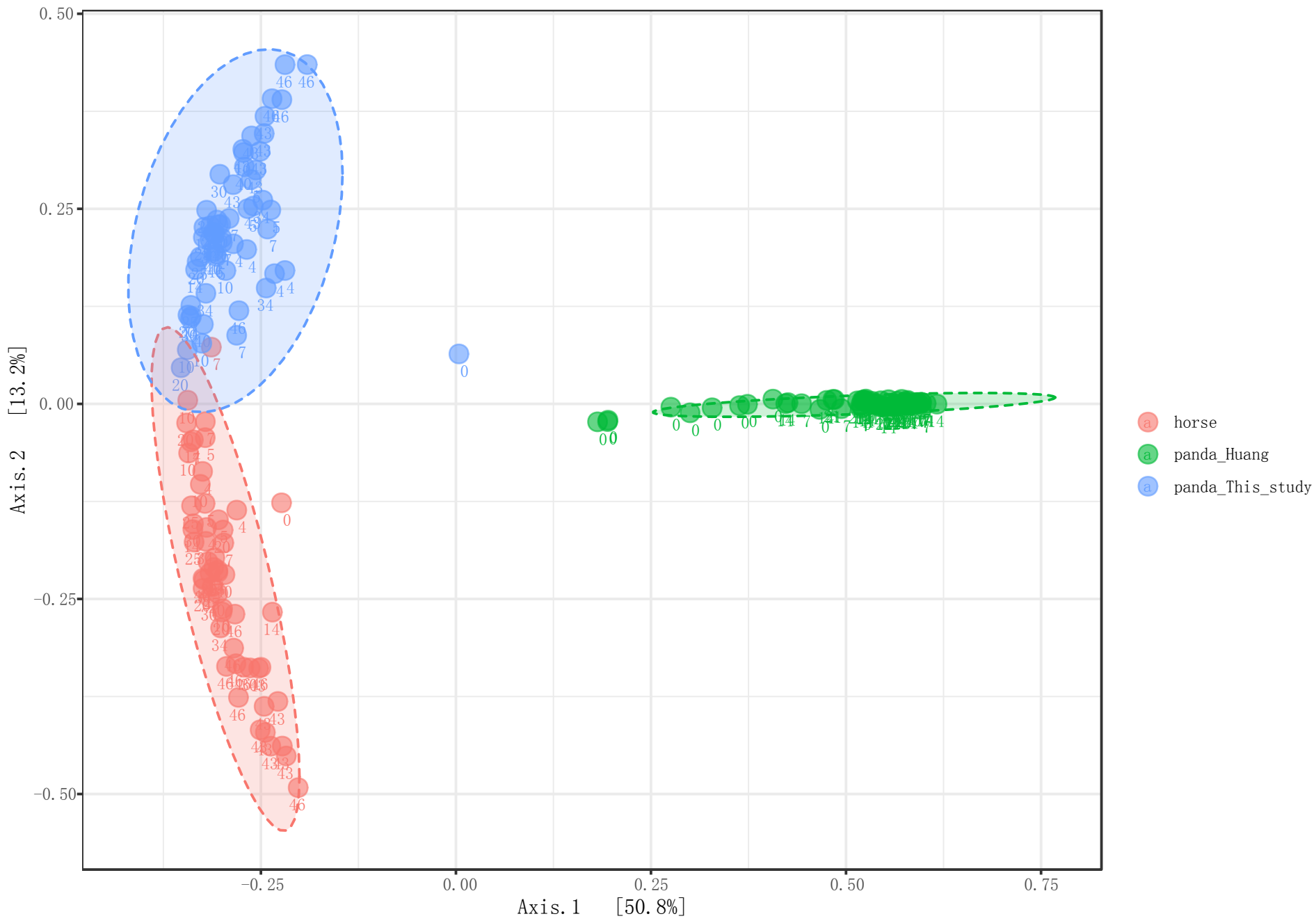

Supplement: SUPPLEMENTARY FIGURE S11 — Hierarchical cluster analysis of identified differential metabolites between panda-mice and horse-mice. Here, the metabolites with negative and positive ion mode were clustered and shades of blue and red represent down-regulation or up-regulation, respectively. [file Data_Sheet_11.PDF]

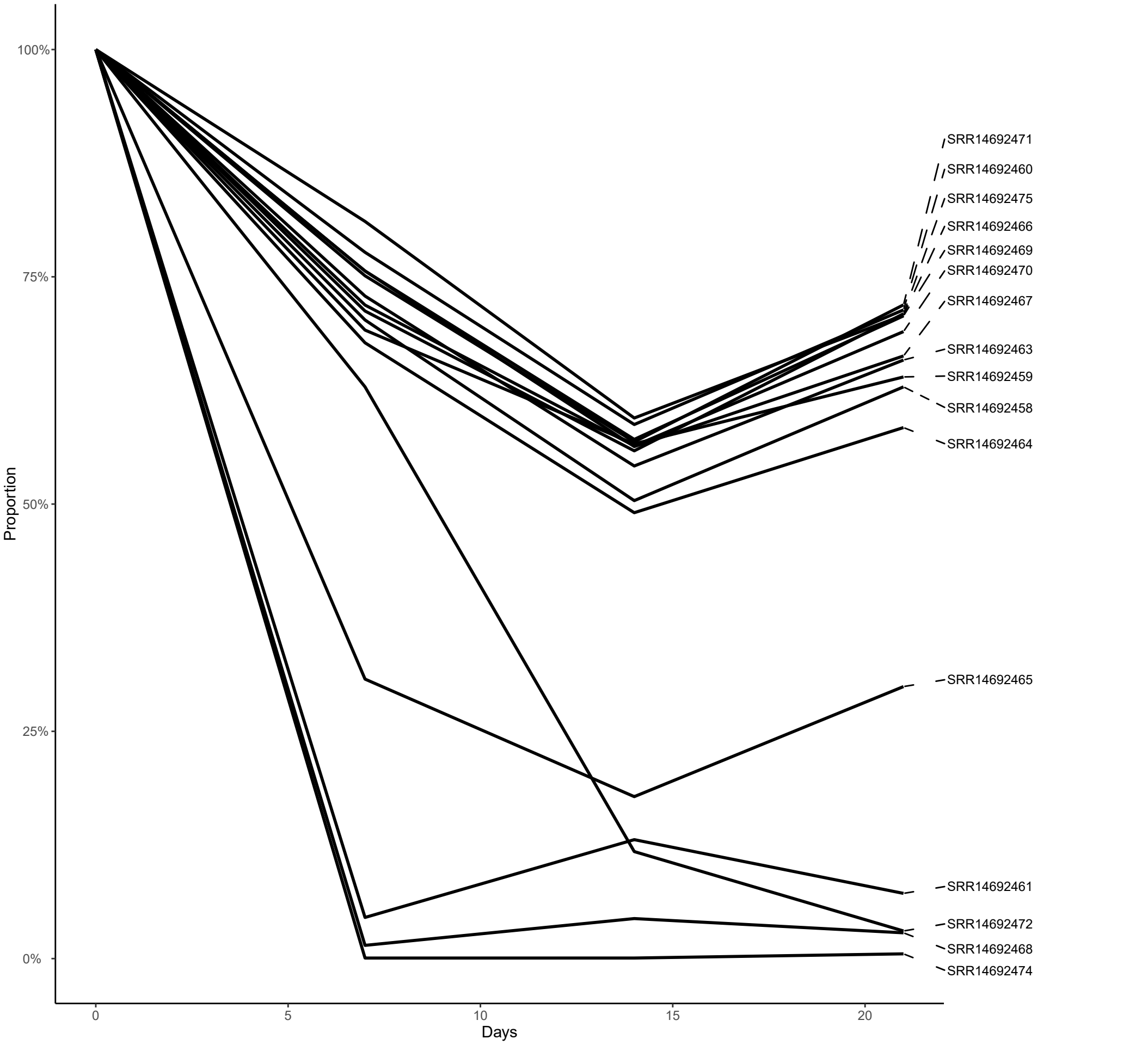

Supplement: SUPPLEMENTARY FIGURE S12 — The network of Spearman correlations of metabolite and bacterial features based on the 16S rRNA gene sequencing results. The type of correlation is indicated. Only significant correlations (p < 0.01) exhibiting an R > 0.5 or R < –0.5 were considered. [file Data_Sheet_12.PDF]

A

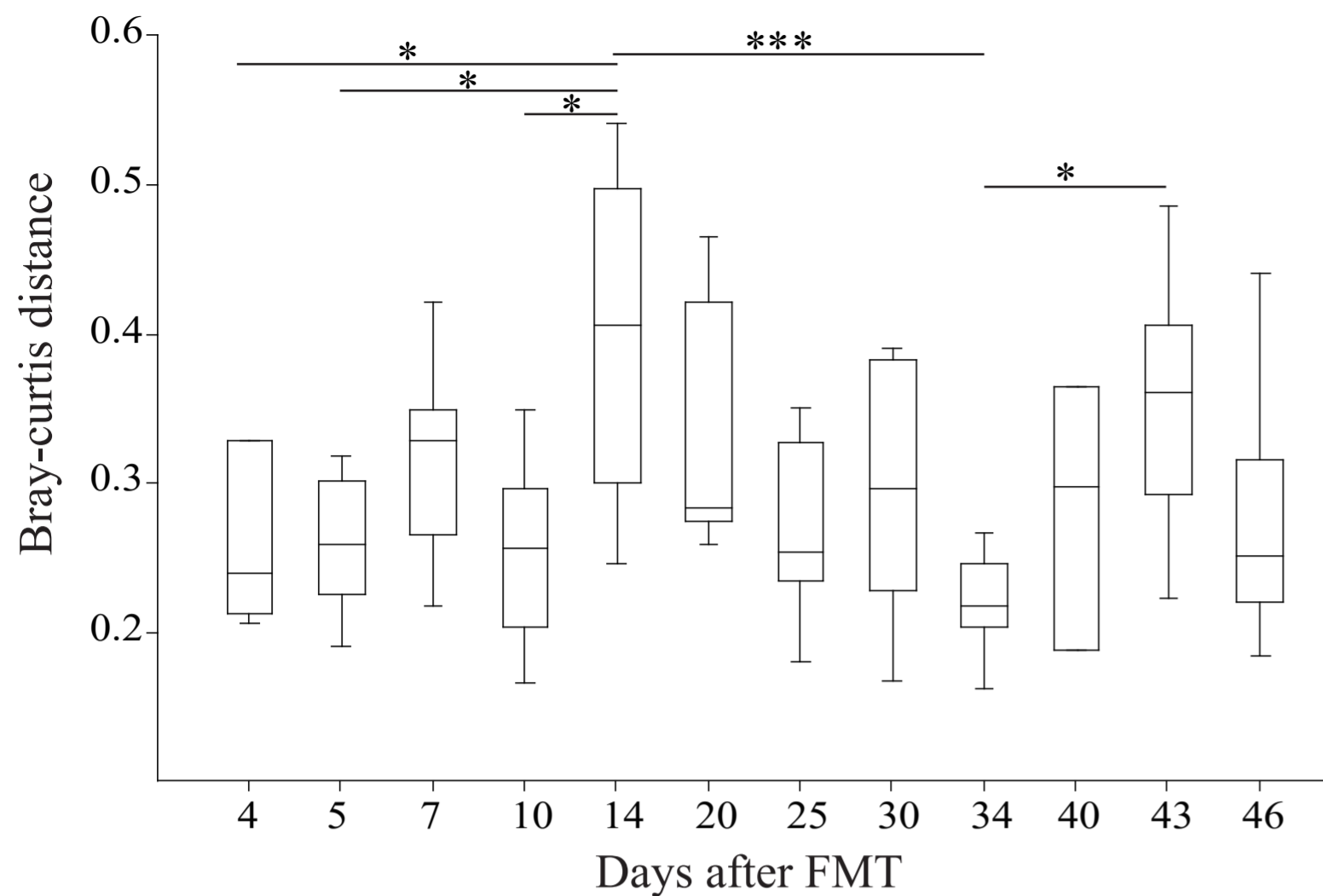

# B

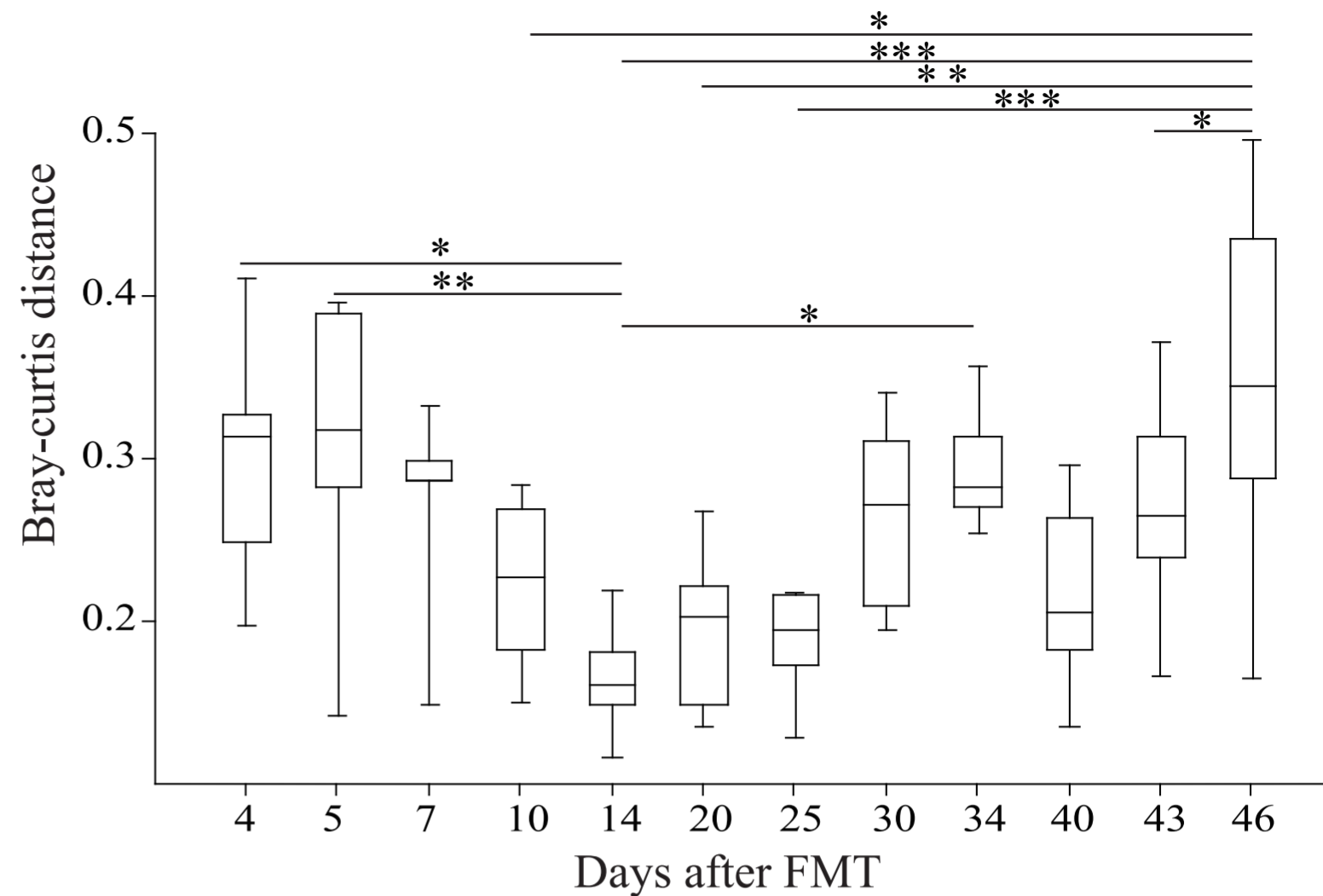

C

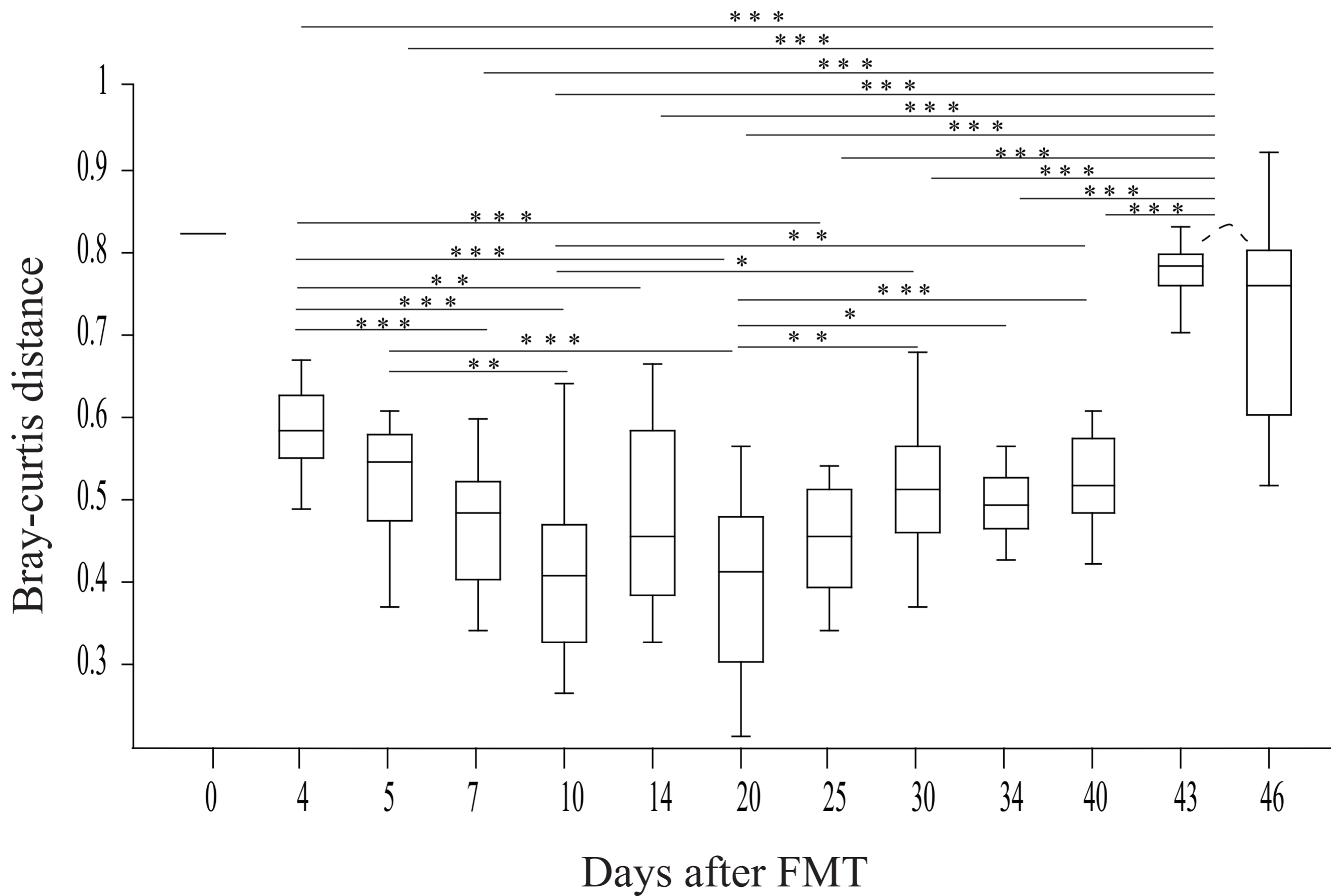

Supplement: SUPPLEMENTARY FIGURE S13 — The PCoA plot with PCo1 and PCo2 based on Bray-Curtis distance of 16S rRNA gene. Panda_Huang showed the samples from Huang et al. (2022). The numbers in this plot showed the time points. [file Data_Sheet_13.PDF]

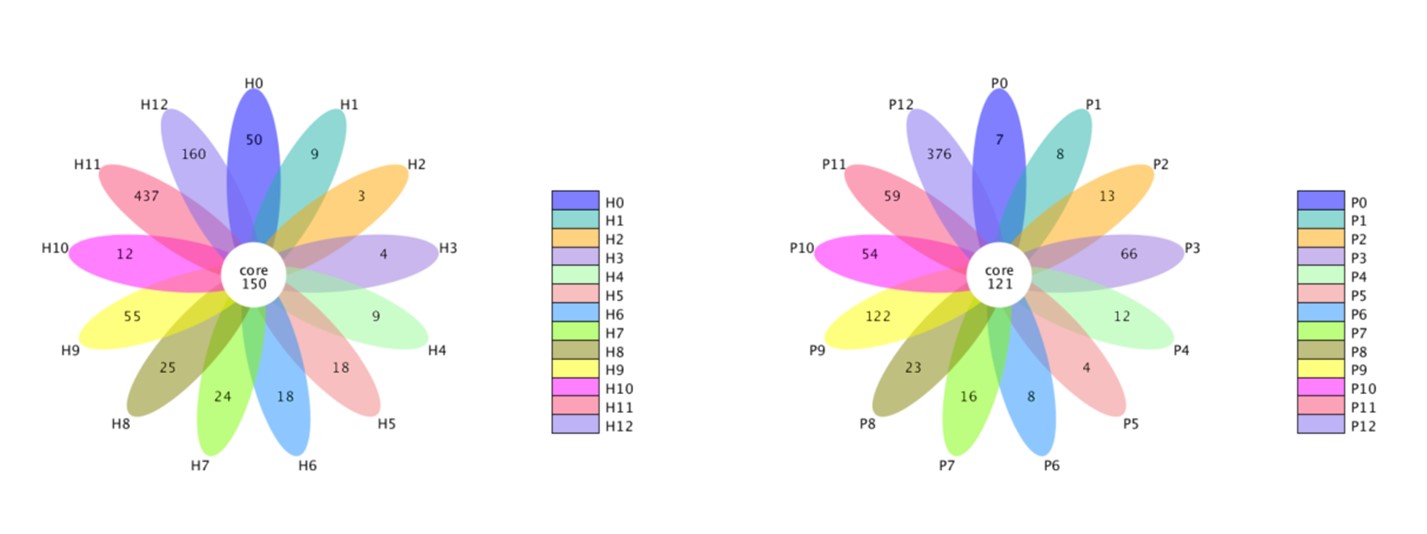

Supplement: SUPPLEMENTARY FIGURE S14 — The changes of average proportions of giant panda fecal microbiota in GF mice at 7th, 14th, and 21st day after FMT. The data came from the reference (Huang et al., 2022). [file Image_1.JPEG]

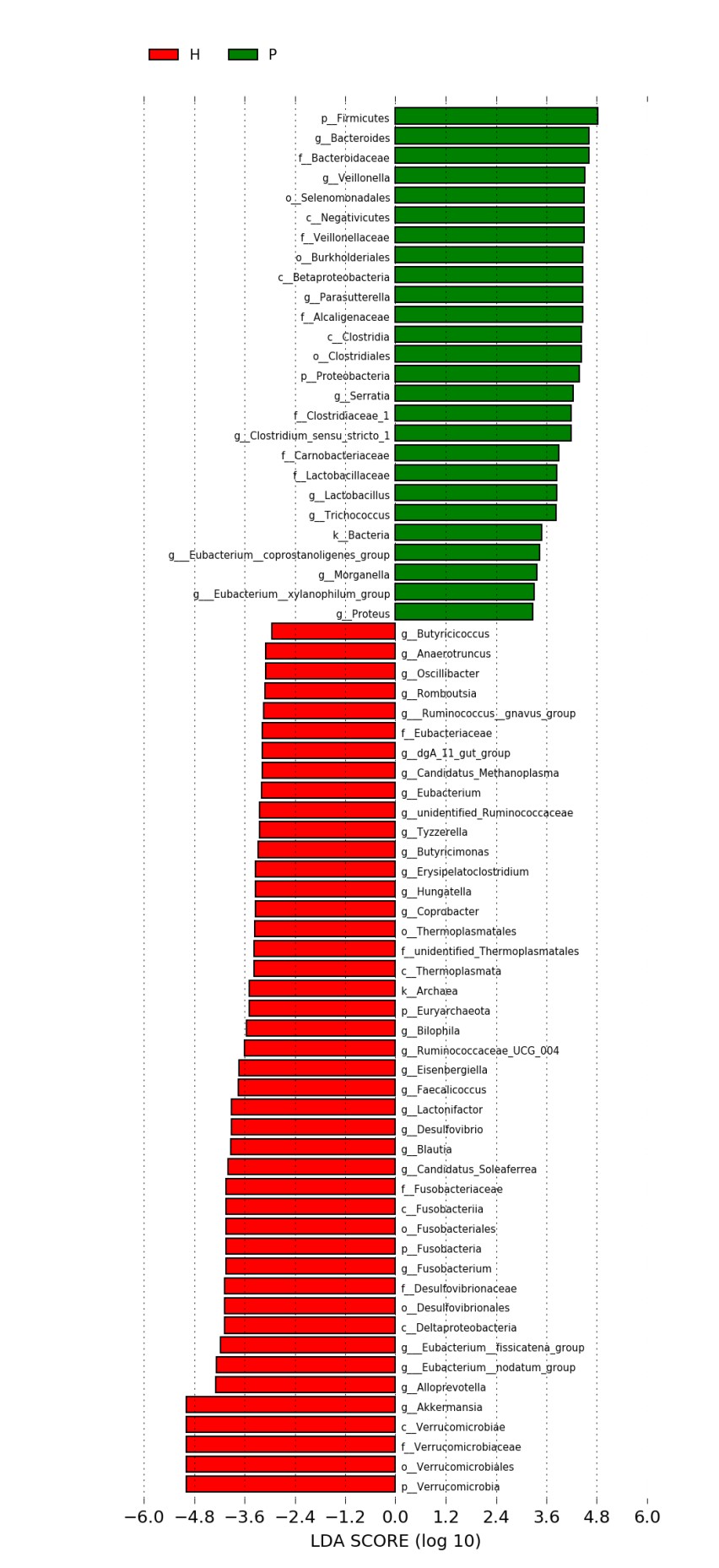

Supplement: SUPPLEMENTARY FIGURE S15 — Comparisons of pairwise Bray-Curtis distances of gut microbiota among GF mice for each time point. (A) The distance among horse-mice; (B) the distance among panda-mice; (C) the distance between horse-mice and panda-mice for corresponding time points. The lines and squares inside boxes represent the median and mean, respectively. Wilcoxon rank-sum test was used in here and *p <0.05, **p <0.01, ***p <0.001. [file Image_2.JPEG]
